# Modelling entomological-climatic interactions of *Plasmodium falciparum *malaria transmission in two Colombian endemic-regions: contributions to a National Malaria Early Warning System

**DOI:** 10.1186/1475-2875-5-66

**Published:** 2006-08-01

**Authors:** Daniel Ruiz, Germán Poveda, Iván D Vélez, Martha L Quiñones, Guillermo L Rúa, Luz E Velásquez, Juan S Zuluaga

**Affiliations:** 1 Escuela de Geociencias y Medio Ambiente, Facultad de Minas, Universidad Nacional de Colombia Sede Medellín, Medellín, Colombia; 2 Programa Ingeniería Ambiental, Escuela de Ingeniería de Antioquia, Calle 25 Sur No. 42–73, Envigado, Antioquia, Colombia; 3 Programa de Estudio y Control de Enfermedades Tropicales, Sede de Investigación Universitaria, Universidad de Antioquia, Medellín, Colombia; 4 Departamento de Salud Pública, Facultad de Medicina, Universidad Nacional de Colombia Sede Bogotá, Bogotá, Colombia; 5 Corporación para Investigaciones Biológicas, Medellín, Colombia

## Abstract

**Background:**

Malaria has recently re-emerged as a public health burden in Colombia. Although the problem seems to be climate-driven, there remain significant gaps of knowledge in the understanding of the complexity of malaria transmission, which have motivated attempts to develop a comprehensive model.

**Methods:**

The mathematical tool was applied to represent *Plasmodium falciparum *malaria transmission in two endemic-areas. Entomological exogenous variables were estimated through field campaigns and laboratory experiments. Availability of breeding places was included towards representing fluctuations in vector densities. Diverse scenarios, sensitivity analyses and instabilities cases were considered during experimentation-validation process.

**Results:**

Correlation coefficients and mean square errors between observed and modelled incidences reached 0.897–0.668 (P > 0.95) and 0.0002–0.0005, respectively. Temperature became the most relevant climatic parameter driving the final incidence. Accordingly, malaria outbreaks are possible during the favourable epochs following the onset of El Niño warm events. Sporogonic and gonotrophic cycles showed to be the entomological key-variables controlling the transmission potential of mosquitoes' population. Simulation results also showed that seasonality of vector density becomes an important factor towards understanding disease transmission.

**Conclusion:**

The model constitutes a promising tool to deepen the understanding of the multiple interactions related to malaria transmission conducive to outbreaks. In the foreseeable future it could be implemented as a tool to diagnose possible dynamical patterns of malaria incidence under several scenarios, as well as a decision-making tool for the early detection and control of outbreaks. The model will be also able to be merged with forecasts of El Niño events to provide a National Malaria Early Warning System.

## Background

The World Health Organization estimates that malaria parasites infect from 200 to 300 million persons and kill more than 1 million people each year, primarily children under the age of five [1]. The efficacy of control measures has decreased over the past decades because mosquitoes and parasites are becoming more resistant to the commonly used insecticides and anti-malarial drugs [2]. As a result, malaria kills more people today than three decades ago. To further complicate matters, the international community suggests that diseases relayed by mosquitoes, such as malaria, are among those infectious diseases most likely to spread dramatically as global temperatures head upward [3,4]. Forecasts from different models suggest that by the end of the 21st century ongoing warming will have enlarged the zone of potential malaria transmission from an area containing 45 percent of the world's population to an area containing about 60 percent [4]. Although this expansion certainly fits the predictions, the cause of that growth may not be attributed convincingly to global warming. Other factors (maybe the dominant contributors) may have been involved as well, for instance: disruption of the environment in ways that favour the mosquitoes' proliferation, declines in vector-control and in other public health programs, and rises in drug and insecticide resistance [4]. To make matters worse, there are a number of 'macro-factors' such as economic inequalities, continuous human migratory patterns and vegetation patterns that may increase or decrease vulnerability and exposure to vector-borne infections and play an important role in regional morbidity and mortality profiles. Clearly, it is impossible to treat climate isolated from the other biological, socioeconomic and demographic contributors if the determination of the potential impact of climate change on malaria incidence is urgently need to be made. However, the hypothesis for a climatic contribution becomes stronger: even though malaria is a highly complex multi-factorial disease, previous studies have identified environmental factors and climate variability as going a considerable way in helping to explain the fluctuations of disease incidence [5-7].

In Colombia, currently, the population exceeds 44 million people; more than five million live in endemic-prone regions. Malaria has recently re-emerged as a significant public health burden in the country: incidence during epidemic years (total positive cases for both *Plasmodium falciparum *and *Plasmodium vivax *malaria scaled by the total population at risk per 1,000 inhabitants) increased from less than 2.5 in 1963 to 6 in 1983 and almost 10 in 1998. During 1996, disease transmission reached 42 cases per 1,000 inhabitants in high-risk areas. In the Chocó Department, on the Colombian Pacific Coast, more than 80,000 cases were reported during 1998, when the population at risk was 380,000 people [8]. During the year 2000 malaria cases were reported in 69% of the 125 towns of the Department of Antioquia, in the Andean region. Besides the aforementioned strongly increasing trend, the Annual Parasite Index also exhibited a significant association between the increase in the number of malaria cases and the occurrence of the El Niño warm event, which is considered the main forcing mechanism of Colombia's hydroclimatology at inter-annual timescales [8]. There is strong evidence that the El Niño event intensifies the annual cycle of malaria cases in endemic rural areas as a consequence of concomitant anomalies in the normal annual cycle of temperature and precipitation. During 'normal' years, endemic malaria exhibits a clear-cut 'normal' annual cycle, which is associated with prevalent climatic conditions. During El Niño events, characterized by increasing temperatures and decreasing monthly rainfalls, river flows and soil moistures, malaria outbreaks were found to be enhanced by climate anomalies. The annual cycle of malaria seems to be affected in its amplitude (increase in the number of cases), although the phase (or timing) remains unaltered in most malaria prone-regions of the country [8].

As recent scientific and technological advances have permitted much better predictions of the El Niño event, there is an evident opportunity to incorporate this climate forecasting capability into programs, campaigns, control measures and mitigation plans to reduce the human health impact of malaria outbreaks in Colombian prone-regions. Nevertheless, there remain significant gaps of knowledge in the understanding of the interactions between climatic factors and the dynamics of malaria transmission, which have motivated attempts to explore and develop mathematical comprehensive models.

Detailed dynamic models of malaria epidemiology have been developed extensively and have made important contributions to understanding the transmission of malaria and other diseases [3,9-21]. There are limitations, however. Although these models can provide adequate approximations to some biological and epidemiological characteristics, most of them have not been able to describe the overall transmission dynamics [21]. Even though mosquitoes and vertebrate host dynamics are generally included, most models do not include changes in environmental and climatic patterns that could affect disease incidence in both spatial and temporal scales, nor do they include the social, economical and demographical conditions prevailing in the communities that were studied. In this article experiences in developing and implementing a vector-borne disease model, which is based on previously proposed differential-equation 'compartment' models, are reported. The aim was to validate the mathematical tool by comparing its results under conditions of intense transmission in two specific malaria prone-regions of Colombia. To do so, malaria prevalence changes were monitored in these areas and model predictions were tested to assess their consistency with field observations.

In developing the mathematical model, three major steps were followed: (i) First, the parasite transmission cycle was studied to define those endogenous variables strongly affected by climatic conditions. (ii) The vector ecology, behavioural patterns of mosquitoes and entomological parameters were analysed in order to determine those exogenous variables (and their relationships with climatic anomalies) that might be relevant for representing malaria transmission. Analyses were focused on the necessity to represent the observed fluctuations in vector density. (iii) The vector population dynamics during pre-imago stages was represented, including the availability of adequate breeding sites and the predator-prey interactions during larval stage.

In applying and validating the mathematical tool, five major steps were followed: (a) homogeneity analysis of hydrological time series, to detect possible changes in mean and variance, as well as significant trends in historical series of both temperature and precipitation. (b) Analysis of total positive malaria cases, to determine the behaviour of epidemiological time series, to establish the initial values of state variables, and for fine-tuning the parameters. (c) Correlation analysis, to determine the contribution of climate factors on the temporal variance of malaria transmission. (d) Simulation-base scenario, to analyse the behaviour of the proposed differential-equation model and its preliminary results. And (e) simulation-alternate scenarios, to analyse several simulation results of the set of non-linear differential equations when perturbations of several exogenous variables, changes in control parameters and changing climate scenarios were considered.

The mathematical tool allowed improving the understanding of the linkages between climate patterns and malaria outbreaks, mainly during the onset of ENSO warm events. Experiences suggest that forecasts of future El Niño events will be able to be merged with mathematical models to provide a Malaria Early Warning System (MEWS) to enhance disease surveillance, control and response to epidemics, and facilitate early, coupled and environmentally sound public health interventions [8].

## Methods

### Main module

**Figure 1:**
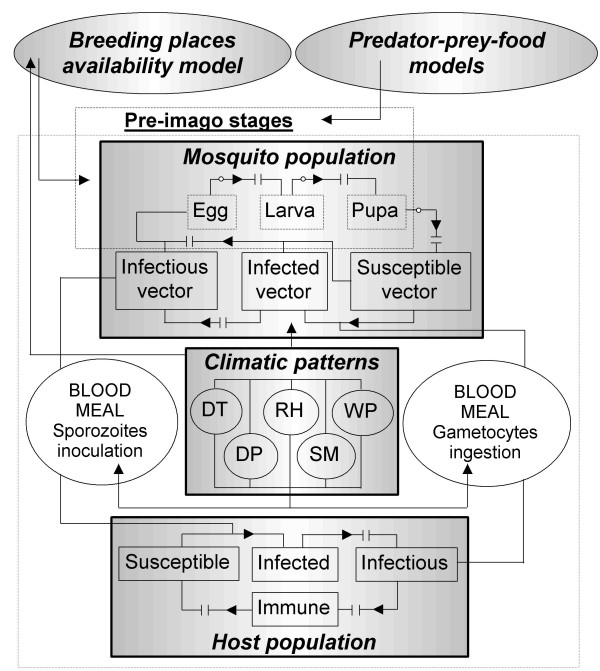
**Schematic diagram**. The main blocks represent linking components: mosquito population (vector ecology), vertebrate host (human) population, and weather patterns. Level variables are represented by small rectangles: egg, larva and pupa (virtual compartments), susceptible vector, infected vector and infectious vector (top), and susceptible, infected, infectious, and immune host (bottom). Mosquito and host populations are linked by the transmission of parasites through mosquito blood meals (vertical). The non-continuous arrows represent time delays. The ovals on top represent: (left) table functions affecting the main module and denoting the availability of adequate breeding sites, and (right) predator-prey interactions during pre-imago stages. On non-continuous arrows, the circles represent hatching, larva development and emergence success. Variables DT, RH, DP, WP and SM represent mean daily temperatures, mean daily relative humidity values, total daily rainfall records, wind patterns, and soil moistures, respectively. DT and HR affect the vector ecology and the blood meal. DP and SM affect the availability of adequate larval habitats. At the first approximation, wind patterns, which may affect mosquito densities, are not considered. The 'virtual' compartments representing pre-imago stages are only used when vector densities obtained by simulation are required for estimating the Vectorial Capacity.

Figure 1 depicts the schematic diagram of the proposed overall malaria model. The comprehensive tool has three linking components that can be simulated separately, if desired, except in the case of vector-host interactions. These components are the human host population, the mosquito population (vector ecology), and the weather patterns. Climate strongly affects the corresponding interaction between populations during a blood meal, as well as the vector ecology and the availability of breeding sites.

By ignoring the secondary *exo-erythrocytic schizogony*, that takes place in the parenchymal cells of the liver, simulation was limited to representing *P. falciparum *malaria transmission. Based upon the widely known biological and epidemiological aspects of malaria transmission, a susceptible person may become a member of the infected class if the individual is inoculated with sporozoites during an adult female *Anopheles *mosquito infective bite. The *exo-erythrocytic schizogony *and the *erythrocytic schizogony *take place in the infected vertebrate host. During this stage, sexually differentiated forms (gametocytes) are present in the host's blood circulation and can be taken by a susceptible female mosquito during a new blood meal. Parasites can then be transmitted from this mosquito to a susceptible person in some latter human-vector interaction. The progressive increase of parasitaemia in the infectious host as a result of multiple *erythrocytic *cycles may be slowed down by the immune response of the human. Finally, the immune individual loses immunity and returns to the reservoir of susceptible hosts.

In the vector ecology, a susceptible adult female *Anopheles *mosquito becomes a member of the infected class when it takes a blood meal from an infectious host and ingests the gametocytes present in the individual's blood stream. The vector finally enters the reservoir of infectious mosquitoes after the completion of the sporogonic cycle.

Since vector density is commonly assumed to be constant by most researchers, this variable has played a minor role in the construction of their mathematical infectious disease models. It is generally accepted that it is very difficult to arrive at a reliable estimate of the changes in vector density over large areas as a result of changes in temperature, precipitation and humidity. The effort of this research is aimed at fully representing the vector density in human-mosquito interactions, by considering three additional stages (virtual compartments) in the mosquito ecology: the pre-imago stages eggs, larvae, and pupae. Interests included the analysis of average development periods from eggs to adult mosquitoes, expressed as time delays and the related survival probabilities, expressed as successes in hatching, larval development, and adult emergence. All these entomological variables seem to be strongly dependent on the ambient temperature or water temperature of the breeding site.

### Breeding places availability model

The availability of larval habitats (Figure 1) has been based on level fluctuations of the quantity of water in breeding places of varying capacities. Table functions affecting the egg laying (oviposition) of a vector population -into the main module- have been estimated through Fuzzy Logic. This methodology was used to generalize the discrete events 'larval habitat completely available-completely full' and 'larval habitat not available-completely dry' to a continuous (fuzzy) form or partial truth. We considered the collections of water as 'reservoirs' ranging from 10 to 60 mm in depth opened to observed rainfall, estimated actual evaporation, and assumed constant infiltration. Daily evapotranspiration was estimated using the models introduced by: Turc (1945), assuming a homogeneous distribution of evapotranspiration throughout the year; Coutagne (1974) using Budyko's equation (1974); Cenicafé (1997), a specific evaporation model suited for Colombia; and Thornthwaite (1948), assuming a relation between actual and potential evapotranspiration of about 0.70 and a homogeneous distribution throughout each month [22].

As ovipositing anopheline females often prefer standing fresh water with low organic contents [23], this model determine the number of successive days that a breeding site remained between 90 and 100% of its capacity. This period of time is needed to complete all stages including egg batching, growth and metamorphosis in aquatic existence, and adult (imago) emergence. A 'favourable' condition is given when this period exceeds 12–15 successive days [2], and is normally affected by the water temperature of the breeding site. Finally, as anopheline eggs cannot survive prolonged desiccation, we assumed that a breeding site is 'not favourable' for the development of the species during pre-imago stages if it remains dry for more than two successive days. For the sake of simplicity, other relevant factors such as exposure to sunlight, emergent vegetation, temperature, salinity, and organic content [23], were not included in the model.

### Predator-prey interaction models

This module allowed us to represent the interaction between the pre-imago stages of the mosquito population (mainly during larvae stage) and natural predators (Figure 1). As a first effort, the Lotka-Volterra predator-prey type model [24-26] has been introduced into the differential-equation system to analyse the dynamics of both the prey and predator populations. Parameters for other types of models that were included in the model, but are not currently used, including Henize predator-prey model [27,9], Kaibab III prey-food model [10], Ecological System with External Forcing [9], and Autonomous Ecological System [9], are being calibrated through laboratory experiments.

### System of coupled non-linear differential equations

The mathematical model represents the feedback mechanisms and interactions depicted in Figure 1. The comprehensive tool is based on a system of coupled non-linear differential equations, which is solved through a fourth order Runge-Kutta numerical algorithm. Simulations are being run using the computer software Powersim Constructor Version 2.51. The system of differential equations for the human population component and hence, the dynamics of malaria transmission in human hosts, is the following:

d(HUS)dt=Rna⋅Phu+γ⋅HUM−Rmo⋅HUS−VC⋅HUFPhu⋅HUS,d(HUI)dt=VC⋅HUFPhu⋅HUS−[HUI]t−(kin+ker)−Rmo⋅HUI,d(HUF)dt=[HUI]t−(kin+ker)−V⋅HUF−Rmo⋅HUF, andd(HUM)dt=V⋅HUF−γ⋅HUM−Rmo⋅HUM.
 MathType@MTEF@5@5@+=feaafiart1ev1aaatCvAUfKttLearuWrP9MDH5MBPbIqV92AaeXatLxBI9gBaebbnrfifHhDYfgasaacH8akY=wiFfYdH8Gipec8Eeeu0xXdbba9frFj0=OqFfea0dXdd9vqai=hGuQ8kuc9pgc9s8qqaq=dirpe0xb9q8qiLsFr0=vr0=vr0dc8meaabaqaciaacaGaaeqabaqabeGadaaakqaabeqaamaalaaabaacbeGae8hzaq2aaeWaceaacqWFibascqWFvbqvcqWFtbWuaiaawIcacaGLPaaaaeaacqWFKbazcqWF0baDaaGaeyypa0Jae8Nuai1aaSbaaSqaaiab=5gaUjab=fgaHbqabaGccqGHflY1cqWFqbaudaWgaaWcbaGae8hAaGMae8xDauhabeaakiabgUcaRGGabiab+n7aNjabgwSixlab=Heaijab=vfavjab=1eanjabgkHiTiab=jfasnaaBaaaleaacqWFTbqBcqWFVbWBaeqaaOGaeyyXICTae8hsaGKae8xvauLae83uamLaeyOeI0Iae8NvayLae83qamKaeyyXIC9aaSaaaeaacqWFibascqWFvbqvcqWFgbGraeaacqWFqbaudaWgaaWcbaGae8hAaGMae8xDauhabeaaaaGccqGHflY1cqWFibascqWFvbqvcqWFtbWucqGGSaalaeaadaWcaaqaaiab=rgaKnaabmGabaGae8hsaGKae8xvauLae8xsaKeacaGLOaGaayzkaaaabaGae8hzaqMae8hDaqhaaiabg2da9iab=zfawjab=neadjabgwSixpaalaaabaGae8hsaGKae8xvauLae8NrayeabaGae8huaa1aaSbaaSqaaiab=HgaOjab=vha1bqabaaaaOGaeyyXICTae8hsaGKae8xvauLae83uamLaeyOeI0YaamWaceaacqWFibascqWFvbqvcqWFjbqsaiaawUfacaGLDbaadaWgaaWcbaGae8hDaqNaeyOeI0YaaeWaceaacqWFRbWAdaWgaaadbaGae8xAaKMae8NBa4gabeaaliabgUcaRiab=TgaRnaaBaaameaacqWFLbqzcqWFYbGCaeqaaaWccaGLOaGaayzkaaaabeaakiabgkHiTiab=jfasnaaBaaaleaacqWFTbqBcqWFVbWBaeqaaOGaeyyXICTae8hsaGKae8xvauLae8xsaKKaeiilaWcabaWaaSaaaeaacqWFKbazdaqadiqaaiab=Heaijab=vfavjab=zeagbGaayjkaiaawMcaaaqaaiab=rgaKjab=rha0baacqGH9aqpdaWadiqaaiab=Heaijab=vfavjab=LeajbGaay5waiaaw2faamaaBaaaleaacqWF0baDcqGHsisldaqadiqaaiab=TgaRnaaBaaameaacqWFPbqAcqWFUbGBaeqaaSGaey4kaSIae83AaS2aaSbaaWqaaiab=vgaLjab=jhaYbqabaaaliaawIcacaGLPaaaaeqaaOGaeyOeI0Iae8NvayLaeyyXICTae8hsaGKae8xvauLae8NrayKaeyOeI0Iae8Nuai1aaSbaaSqaaiab=1gaTjab=9gaVbqabaGccqGHflY1cqWFibascqWFvbqvcqWFgbGrcqGGSaalcqqGGaaicqqGHbqycqqGUbGBcqqGKbazaeaadaWcaaqaaiab=rgaKnaabmGabaGae8hsaGKae8xvauLae8xta0eacaGLOaGaayzkaaaabaGae8hzaqMae8hDaqhaaiabg2da9iab=zfawjabgwSixlab=Heaijab=vfavjab=zeagjabgkHiTiab+n7aNjabgwSixlab=Heaijab=vfavjab=1eanjabgkHiTiab=jfasnaaBaaaleaacqWFTbqBcqWFVbWBaeqaaOGaeyyXICTae8hsaGKae8xvauLae8xta0KaeiOla4caaaa@FCA0@

The variables HUS, HUI, HUF, and HUM denote the total number of susceptible, malaria-infected, infectious, and immune human hosts at a given time t, respectively. In this component, Martens' equations [3] were followed, although considering the infective stage of the human hosts.

For the vector ecology component, the model uses the following system of coupled non-linear differential equations to describe the dynamics of the pre-imago stages of the vector population:

d(E)dtT1(fL)⋅fL⋅fL_S⋅〈(VS+VI+VF)⋅Rpo〉FI−EkE−E⋅μE⋅T2(E),d(L)dt=EkE−LkL−L⋅μL⋅T3(L)−aS⋅L⋅PD, andd(PU)dt=LkL−PU⋅μPU⋅T4(PU)−PUkem.
 MathType@MTEF@5@5@+=feaafiart1ev1aaatCvAUfKttLearuWrP9MDH5MBPbIqV92AaeXatLxBI9gBaebbnrfifHhDYfgasaacH8akY=wiFfYdH8Gipec8Eeeu0xXdbba9frFj0=OqFfea0dXdd9vqai=hGuQ8kuc9pgc9s8qqaq=dirpe0xb9q8qiLsFr0=vr0=vr0dc8meaabaqaciaacaGaaeqabaqabeGadaaakqaabeqaamaalaaabaacbeGae8hzaq2aaeWaceaacqWFfbqraiaawIcacaGLPaaaaeaacqWFKbazcqWF0baDaaGae8hvaq1aaSbaaSqaaiab=fdaXaqabaGcdaqadiqaaiab=zgaMnaaBaaaleaacqWFmbataeqaaaGccaGLOaGaayzkaaGaeyyXICTae8Nzay2aaSbaaSqaaiab=XeambqabaGccqGHflY1cqWFMbGzdaWgaaWcbaGae8htaWKaei4xa8Lae83uamfabeaakiabgwSixpaaamqabaWaaeWaceaacqWFwbGvcqWFtbWucqGHRaWkcqWFwbGvcqWFjbqscqGHRaWkcqWFwbGvcqWFgbGraiaawIcacaGLPaaacqGHflY1cqWFsbGudaWgaaWcbaGae8hCaaNae83Ba8gabeaaaOGaayzkJiaawQYiamaaBaaaleaacqWFgbGrcqWFjbqsaeqaaOGaeyOeI0YaaSaaaeaacqWFfbqraeaacqWFRbWAdaWgaaWcbaGae8xraueabeaaaaGccqGHsislcqWFfbqrcqGHflY1iiqacqGF8oqBdaWgaaWcbaGae8xraueabeaakiabgwSixlab=rfaunaaBaaaleaacqWFYaGmaeqaaOWaaeWaceaacqWFfbqraiaawIcacaGLPaaacqGGSaalaeaadaWcaaqaaiab=rgaKnaabmGabaGae8htaWeacaGLOaGaayzkaaaabaGae8hzaqMae8hDaqhaaiabg2da9maalaaabaGae8xraueabaGae83AaS2aaSbaaSqaaiab=veafbqabaaaaOGaeyOeI0YaaSaaaeaacqWFmbataeaacqWFRbWAdaWgaaWcbaGae8htaWeabeaaaaGccqGHsislcqWFmbatcqGHflY1cqGF8oqBdaWgaaWcbaGae8htaWeabeaakiabgwSixlab=rfaunaaBaaaleaacqWFZaWmaeqaaOWaaeWaceaacqWFmbataiaawIcacaGLPaaacqGHsislcqWFHbqydaWgaaWcbaGae83uamfabeaakiabgwSixlab=XeamjabgwSixlab=bfaqjab=reaejabcYcaSiabbccaGiabbggaHjabb6gaUjabbsgaKbqaamaalaaabaGae8hzaq2aaeWaceaacqWFqbaucqWFvbqvaiaawIcacaGLPaaaaeaacqWFKbazcqWF0baDaaGaeyypa0ZaaSaaaeaacqWFmbataeaacqWFRbWAdaWgaaWcbaGae8htaWeabeaaaaGccqGHsislcqWFqbaucqWFvbqvcqGHflY1cqGF8oqBdaWgaaWcbaGae8huaaLae8xvaufabeaakiabgwSixlab=rfaunaaBaaaleaacqWF0aanaeqaaOWaaeWaceaacqWFqbaucqWFvbqvaiaawIcacaGLPaaacqGHsisldaWcaaqaaiab=bfaqjab=vfavbqaaiab=TgaRnaaBaaaleaacqWFLbqzcqWFTbqBaeqaaaaakiabc6caUaaaaa@C8DC@

The dynamic variables E, L, and PU denote the total number of eggs, larvae, and pupae at a given time t, respectively. The table functions T_1_, T_2_, T_3_, and T_4 _represent the multiplier factors affecting oviposition (competition during egg-laying), and eggs, larvae and pupae mortalities, respectively.

To describe the dynamics of the imago stages of the vector population and hence, the dynamics of malaria transmission in adult female mosquitoes, the model uses a second set of differential equations:

d(VS)dt=PUkem−(μm+αm)⋅VS−VS⋅f⋅HUFPhu⋅S_V,d(VI)dt=VS⋅f⋅HUFPhu⋅S_V−(μm+αm)⋅VI−VIn, andd(VF)dt=VIn−(μm+αm)⋅VF.
 MathType@MTEF@5@5@+=feaafiart1ev1aaatCvAUfKttLearuWrP9MDH5MBPbIqV92AaeXatLxBI9gBaebbnrfifHhDYfgasaacH8akY=wiFfYdH8Gipec8Eeeu0xXdbba9frFj0=OqFfea0dXdd9vqai=hGuQ8kuc9pgc9s8qqaq=dirpe0xb9q8qiLsFr0=vr0=vr0dc8meaabaqaciaacaGaaeqabaqabeGadaaakqaabeqaamaalaaabaacbeGae8hzaq2aaeWaceaacqWFwbGvcqWFtbWuaiaawIcacaGLPaaaaeaacqWFKbazcqWF0baDaaGaeyypa0ZaaSaaaeaacqWFqbaucqWFvbqvaeaacqWFRbWAdaWgaaWcbaGae8xzauMae8xBa0gabeaaaaGccqGHsisldaqadiqaaGGabiab+X7aTnaaBaaaleaacqWFTbqBaeqaaOGaey4kaSIae4xSde2aaSbaaSqaaiab=1gaTbqabaaakiaawIcacaGLPaaacqGHflY1cqWFwbGvcqWFtbWucqGHsislcqWFwbGvcqWFtbWucqGHflY1cqWFMbGzcqGHflY1daWcaaqaaiab=Heaijab=vfavjab=zeagbqaaiab=bfaqnaaBaaaleaacqWFObaAcqWF1bqDaeqaaaaakiabgwSixlab=nfatjabc+faFjab=zfawjabcYcaSaqaamaalaaabaGae8hzaq2aaeWaceaacqWFwbGvcqWFjbqsaiaawIcacaGLPaaaaeaacqWFKbazcqWF0baDaaGaeyypa0Jae8NvayLae83uamLaeyyXICTae8NzayMaeyyXIC9aaSaaaeaacqWFibascqWFvbqvcqWFgbGraeaacqWFqbaudaWgaaWcbaGae8hAaGMae8xDauhabeaaaaGccqGHflY1cqWFtbWucqGGFbWxcqWFwbGvcqGHsisldaqadiqaaiab+X7aTnaaBaaaleaacqWFTbqBaeqaaOGaey4kaSIae4xSde2aaSbaaSqaaiab=1gaTbqabaaakiaawIcacaGLPaaacqGHflY1cqWFwbGvcqWFjbqscqGHsisldaWcaaqaaiab=zfawjab=Leajbqaaiab=5gaUbaacqGGSaalcqqGGaaicqqGHbqycqqGUbGBcqqGKbazaeaadaWcaaqaaiab=rgaKnaabmGabaGae8NvayLae8NrayeacaGLOaGaayzkaaaabaGae8hzaqMae8hDaqhaaiabg2da9maalaaabaGae8NvayLae8xsaKeabaGae8NBa4gaaiabgkHiTmaabmGabaGae4hVd02aaSbaaSqaaiab=1gaTbqabaGccqGHRaWkcqGFXoqydaWgaaWcbaGae8xBa0gabeaaaOGaayjkaiaawMcaaiabgwSixlab=zfawjab=zeagjabc6caUaaaaa@B589@

The dynamic variables VS, VI, and VF represent the susceptible or non-infectious, malaria-infected, and malaria infectious mosquito (adult females) population sizes at a given time t, respectively.

To represent the dynamics of natural predators, the following equation is initially considered in the overall malaria model:

d(PD)dt=bp⋅L⋅PD−mp⋅PD.
 MathType@MTEF@5@5@+=feaafiart1ev1aaatCvAUfKttLearuWrP9MDH5MBPbIqV92AaeXatLxBI9gBaebbnrfifHhDYfgasaacH8akY=wiFfYdH8Gipec8Eeeu0xXdbba9frFj0=OqFfea0dXdd9vqai=hGuQ8kuc9pgc9s8qqaq=dirpe0xb9q8qiLsFr0=vr0=vr0dc8meaabaqaciaacaGaaeqabaqabeGadaaakeaadaWcaaqaaGqabiab=rgaKnaabmGabaGae8huaaLae8hraqeacaGLOaGaayzkaaaabaGae8hzaqMae8hDaqhaaiabg2da9iab=jgaInaaBaaaleaacqWFWbaCaeqaaOGaeyyXICTae8htaWKaeyyXICTae8huaaLae8hraqKaeyOeI0Iae8xBa02aaSbaaSqaaiab=bhaWbqabaGccqGHflY1cqWFqbaucqWFebarcqGGUaGlaaa@499E@

The exogenous and endogenous variables considered above are described in Tables 1 and 2 for the mosquito population, Tables 3 and 4 for the human population, and Tables 5 and 6 for the represented infectious disease models (Vectorial Capacity [28], Entomological Inoculation Rate [29], and Basic Reproduction Rate [3]). Five assumptions on vector density, denoted by m, have been used for calibration (see parameter K1 in Table 6): (a) a Human Biting Rate observed during field campaigns for both indoor and outdoor landing captures (HBD); (b) a vector density obtained through simulation and considering the dynamics of mosquito ecology (DC); (c) a critical density threshold necessary to maintain parasite transmission [15] (DCR); (d) an assumed constant density (D_0); and (e) a variable vector density considering seasonal fluctuations (D_1).

**Table 1:** Exogenous variables considered for mosquito population

**Exogenous variable**	**Used variable**		**Depending on**	**Default value**
Hatching time delay	k_E _or R_E	[days]	Water T	(A)
Eggs becoming non-viable	φ_E _or E_NV	[days]	Water T	(B)
Larvae developing time delay	k_L _or R_L	[days]	Water T	(C)
Larvae becoming non-viable	φ_L _or L_NV	[days]	Water T	(D)
Adult emergence time delay	k_em _or R_EM	[days]	Water T	(E)
Pupae becoming non-viable	φ_PU_, PU_NV	[days]	Water T	(F)
Induced mortality of mosquitoes	IM_M	[days]	SEC	98–191.8 [21]
*Plasmodium *species				*P. falciparum*
Human Blood Index	HBI	[dec]	Species	0.38–0.46 [3]
Rate of oviposition (eggs per batch)	R_po _or R_O	[eggs/vector]	T, SEC	75–150
Degree days required for digestion of blood	D_bd_	[°C-day]	RH	36.5 at RH 70–80% [3]
Minimum T required for digestion of blood meal	T_min, bd_	[°]		9.9 [3]
Degree days required for parasite development	D_m_	[°-day]	Species	100–120 [3]
Min T required for parasite development	T_min, p_	[°]	Species	16 [3]

(A) k_E _represents the average period of time spent in the virtual compartment E (eggs) and denotes the time elapsed between oviposition and the development into larva-1st instar. Values of k_E _range from 1 1/2 days at 30° to 7 days at 20°, and were estimated under controlled laboratory conditions (CLC) for temperatures of 24, 27, 30, and 33°C. (B) Values of φ_E _range from 5 days for temperatures ≤23°C and ≥31°C, to 50 days at 27°C (under CLC). (C) k_L _represents the average period of time spent in the virtual compartment L (larvae) and denotes the time elapsed since the eclosion (or larva-1st instar) until the development into pupa. Values of k_L _may range from 8.9 days at 33°C to 13.6 days at 24°C (under CLC). (D) Values of φ_L _range from 3 days for temperatures ≤23°C and ≥31°C, to 32 days at 27°C (under CLC). (E) k_em _represents the average period of time spent in the virtual compartment PU (pupae) and denotes the time elapsed between pupation and the emergence of an adult. Values of k_em _range from 1 day at 33°C to 2 days at 24°C (under CLC). Finally, (F) values of φ_PU _range from 4 days for temperatures ≤23°C and ≥30°C, to 11 days at 27°C (under CLC). For more detail, see Figure 10B. The actual average temperature T must range between T_min, bd _and the maximum temperature of about 40°C (this relationship was estimated for *An. maculipennis *and is used because it agrees with the observations of other *Anopheles *species [36,17,37,30,38]).

**Table 2:** Endogenous variables considered for mosquito population

**Endogenous variable**	**Used variable**		**Depending on**	**Function**
Rate of eggs becoming non-viable	μ_E _or Mu_E	[1/day]	φ_E_	1φE MathType@MTEF@5@5@+=feaafiart1ev1aaatCvAUfKttLearuWrP9MDH5MBPbIqV92AaeXatLxBI9gBaebbnrfifHhDYfgasaacH8akY=wiFfYdH8Gipec8Eeeu0xXdbba9frFj0=OqFfea0dXdd9vqai=hGuQ8kuc9pgc9s8qqaq=dirpe0xb9q8qiLsFr0=vr0=vr0dc8meaabaqaciaacaGaaeqabaqabeGadaaakeaadaWcaaqaaGqabiab=fdaXaqaaGGabiab+z8aMnaaBaaaleaacqWFfbqraeqaaaaaaaa@30A6@
Rate of larvae becoming non-viable	μ_L _or Mu_L	[1/day]	φ_L_	1φL MathType@MTEF@5@5@+=feaafiart1ev1aaatCvAUfKttLearuWrP9MDH5MBPbIqV92AaeXatLxBI9gBaebbnrfifHhDYfgasaacH8akY=wiFfYdH8Gipec8Eeeu0xXdbba9frFj0=OqFfea0dXdd9vqai=hGuQ8kuc9pgc9s8qqaq=dirpe0xb9q8qiLsFr0=vr0=vr0dc8meaabaqaciaacaGaaeqabaqabeGadaaakeaadaWcaaqaaGqabiab=fdaXaqaaGGabiab+z8aMnaaBaaaleaacqWFmbataeqaaaaaaaa@30B4@
Rate of pupae becoming non-viable	μ_PU _or Mu_PU	[1/day]	φ_PU_	1φPU MathType@MTEF@5@5@+=feaafiart1ev1aaatCvAUfKttLearuWrP9MDH5MBPbIqV92AaeXatLxBI9gBaebbnrfifHhDYfgasaacH8akY=wiFfYdH8Gipec8Eeeu0xXdbba9frFj0=OqFfea0dXdd9vqai=hGuQ8kuc9pgc9s8qqaq=dirpe0xb9q8qiLsFr0=vr0=vr0dc8meaabaqaciaacaGaaeqabaqabeGadaaakeaadaWcaaqaaGqabiab=fdaXaqaaGGabiab+z8aMnaaBaaaleaacqWFqbaucqWFvbqvaeqaaaaaaaa@31EB@
Daily survival probability	p or P_S	[dec]	T	e−1(−4.4+1.31*T−0.03*T2) MathType@MTEF@5@5@+=feaafiart1ev1aaatCvAUfKttLearuWrP9MDH5MBPbIqV92AaeXatLxBI9gBaebbnrfifHhDYfgasaacH8akY=wiFfYdH8Gipec8Eeeu0xXdbba9frFj0=OqFfea0dXdd9vqai=hGuQ8kuc9pgc9s8qqaq=dirpe0xb9q8qiLsFr0=vr0=vr0dc8meaabaqaciaacaGaaeqabaqabeGadaaakeaaieqacqWFLbqzdaahaaWcbeqaaiabgkHiTmaalaaabaGae8xmaedabaWaaeWaceaacqGHsislcqWF0aancqGGUaGlcqWF0aancqGHRaWkcqWFXaqmcqGGUaGlcqWFZaWmcqWFXaqmcqGGQaGkcqWFubavcqGHsislcqWFWaamcqGGUaGlcqWFWaamcqWFZaWmcqGGQaGkcqWFubavdaahaaadbeqaaiab=jdaYaaaaSGaayjkaiaawMcaaaaaaaaaaa@4385@ [3] or longevity under CLC.
Natural mortality rate of mosquitoes	μ_m _or Mu_M	[1/day]	p	**1 - p**
Induced mortality rate of mosquitoes	α_m _or Alfa_M	[1/day]	IM_M	1IM_M MathType@MTEF@5@5@+=feaafiart1ev1aaatCvAUfKttLearuWrP9MDH5MBPbIqV92AaeXatLxBI9gBaebbnrfifHhDYfgasaacH8akY=wiFfYdH8Gipec8Eeeu0xXdbba9frFj0=OqFfea0dXdd9vqai=hGuQ8kuc9pgc9s8qqaq=dirpe0xb9q8qiLsFr0=vr0=vr0dc8meaabaqaciaacaGaaeqabaqabeGadaaakeaadaWcaaqaaGqabiab=fdaXaqaaiab=Leajjab=1eanjabc+faFjab=1eanbaaaaa@3248@
Feeding interval	FI	[days]	T, D_bd_, T_min, bd_	DbdT−Tmin,bd MathType@MTEF@5@5@+=feaafiart1ev1aaatCvAUfKttLearuWrP9MDH5MBPbIqV92AaeXatLxBI9gBaebbnrfifHhDYfgasaacH8akY=wiFfYdH8Gipec8Eeeu0xXdbba9frFj0=OqFfea0dXdd9vqai=hGuQ8kuc9pgc9s8qqaq=dirpe0xb9q8qiLsFr0=vr0=vr0dc8meaabaqaciaacaGaaeqabaqabeGadaaakeaadaWcaaqaaGqabiab=reaenaaBaaaleaacqWFIbGycqWFKbazaeqaaaGcbaGae8hvaqLaeyOeI0Iae8hvaq1aaSbaaSqaaiab=1gaTjab=LgaPjab=5gaUjabcYcaSiab=jgaIjab=rgaKbqabaaaaaaa@3B9F@ [35,31] or length of the gonotrophic cycle under CLC.
*Sporogonic *cycle	n or EIP	[days]	T, D_m_, T_min, p_	DmT−Tmin,p MathType@MTEF@5@5@+=feaafiart1ev1aaatCvAUfKttLearuWrP9MDH5MBPbIqV92AaeXatLxBI9gBaebbnrfifHhDYfgasaacH8akY=wiFfYdH8Gipec8Eeeu0xXdbba9frFj0=OqFfea0dXdd9vqai=hGuQ8kuc9pgc9s8qqaq=dirpe0xb9q8qiLsFr0=vr0=vr0dc8meaabaqaciaacaGaaeqabaqabeGadaaakeaadaWcaaqaaGqabiab=reaenaaBaaaleaacqWFTbqBaeqaaaGcbaGae8hvaqLaeyOeI0Iae8hvaq1aaSbaaSqaaiab=1gaTjab=LgaPjab=5gaUjabcYcaSiab=bhaWbqabaaaaaaa@3937@ or length of the sporogonic period under CLC.
Frequency with which human blood meals are taken	a	[1/day]	FI, HBI, Risk	HBIFI MathType@MTEF@5@5@+=feaafiart1ev1aaatCvAUfKttLearuWrP9MDH5MBPbIqV92AaeXatLxBI9gBaebbnrfifHhDYfgasaacH8akY=wiFfYdH8Gipec8Eeeu0xXdbba9frFj0=OqFfea0dXdd9vqai=hGuQ8kuc9pgc9s8qqaq=dirpe0xb9q8qiLsFr0=vr0=vr0dc8meaabaqaciaacaGaaeqabaqabeGadaaakeaadaWcaaqaaGqabiab=Heaijab=jeacjab=Leajbqaaiab=zeagjab=Leajbaaaaa@3223@

The incubation period of the parasite in the malaria transmission vector n (also called the Extrinsic Incubation Period EIP, *Sporogony*, Exogenous Sexual Phase, or Single Sexual Multiplication in the mosquito host) represents the length of the interval between infection (gametocyte ingestion) and the onset of infectivity (sporozoite migration) in the vector host. This parameter is strongly dependent on actual air average temperature. It may range from 8 days at 31°C to 22 days at 20°C (the mean value commonly used reaches 15 days). The frequency with which human blood meals are taken (a) could also be a function of the representative risks of malaria transmission: for a community under a low risk of malaria (RISK = 0), a = 0.07; for a community under an intermediate risk of malaria (RISK = 1), a= 0.25; and for a community which is under a high-risk of malaria (RISK = 2), a= 0.90 [21].

**Table 3:** Exogenous variables considered for the human population

**Exogenous variable**	**Used variable**		**Depending on**	**Default value**
Natural per-capita natality rate	R_na_	[/day]	SEC	For simplicities' sake, R_na _equals the natural death rate
Natural per-capita death rate	R_mo_	[/day]	SEC	1/(52.5*365) for intermediate conditions
Average infected period	ν	[years]	Parasite species	0.9–1.5 for *Plasmodium falciparum*; 3 for *Plasmodium vivax*
Mean duration of immunity	τ	[years]		1.5

**Table 4:** Endogenous variables considered for human population

**Endogenous variable**	**Used variable**		**Depending on**	**Function**
Total human population at risk	P_hu_	[individual]	HUS, HUI, HUF, HUM	**HUS **+ **HUI **+ **HUF **+ **HUM**
Primary *exo-erythrocytic schizogony*	k_in_	[days]	Parasite species, SEC	5–13 for *P. falciparum*; 2 for *P. vivax*
*Erythrocytic schizogony*	k_er_	[days]	Parasite species, SEC	2 for *P. falciparum*; 2 for *P. vivax*
Average infectious period	1/V or P_F	[years]	HUF, ν, VC, P_hu_	EXP[VC*HUFPhu*ν]−1VC*HUFPhu MathType@MTEF@5@5@+=feaafiart1ev1aaatCvAUfKttLearuWrP9MDH5MBPbIqV92AaeXatLxBI9gBaebbnrfifHhDYfgasaacH8akY=wiFfYdH8Gipec8Eeeu0xXdbba9frFj0=OqFfea0dXdd9vqai=hGuQ8kuc9pgc9s8qqaq=dirpe0xb9q8qiLsFr0=vr0=vr0dc8meaabaqaciaacaGaaeqabaqabeGadaaakeaadaWcaaqaaGqabiab=veafjab=Hfayjab=bfaqnaadmGabaGae8NvayLae83qamKaeiOkaOYaaSaaaeaacqWFibascqWFvbqvcqWFgbGraeaacqWFqbaudaWgaaWcbaGae8hAaGMae8xDauhabeaaaaGccqGGQaGkiiqacqGF9oGBaiaawUfacaGLDbaacqGHsislcqWFXaqmaeaacqWFwbGvcqWFdbWqcqGGQaGkdaWcaaqaaiab=Heaijab=vfavjab=zeagbqaaiab=bfaqnaaBaaaleaacqWFObaAcqWF1bqDaeqaaaaaaaaaaa@4BBE@ [3]
Average immune period	1/γ or P_M	[years]	HUF, τ, VC, P_hu_	EXP[VC*HUFPhu*τ]−1VC*HUFPhu MathType@MTEF@5@5@+=feaafiart1ev1aaatCvAUfKttLearuWrP9MDH5MBPbIqV92AaeXatLxBI9gBaebbnrfifHhDYfgasaacH8akY=wiFfYdH8Gipec8Eeeu0xXdbba9frFj0=OqFfea0dXdd9vqai=hGuQ8kuc9pgc9s8qqaq=dirpe0xb9q8qiLsFr0=vr0=vr0dc8meaabaqaciaacaGaaeqabaqabeGadaaakeaadaWcaaqaaGqabiab=veafjab=Hfayjab=bfaqnaadmGabaGae8NvayLae83qamKaeiOkaOYaaSaaaeaacqWFibascqWFvbqvcqWFgbGraeaacqWFqbaudaWgaaWcbaGae8hAaGMae8xDauhabeaaaaGccqGGQaGkiiqacqGFepaDaiaawUfacaGLDbaacqGHsislcqWFXaqmaeaacqWFwbGvcqWFdbWqcqGGQaGkdaWcaaqaaiab=Heaijab=vfavjab=zeagbqaaiab=bfaqnaaBaaaleaacqWFObaAcqWF1bqDaeqaaaaaaaaaaa@4BCB@ [3]
Prevalence**	G or Prev	[dec]	HUF, P_hu_	HUFPhu MathType@MTEF@5@5@+=feaafiart1ev1aaatCvAUfKttLearuWrP9MDH5MBPbIqV92AaeXatLxBI9gBaebbnrfifHhDYfgasaacH8akY=wiFfYdH8Gipec8Eeeu0xXdbba9frFj0=OqFfea0dXdd9vqai=hGuQ8kuc9pgc9s8qqaq=dirpe0xb9q8qiLsFr0=vr0=vr0dc8meaabaqaciaacaGaaeqabaqabeGadaaakeaadaWcaaqaaGqabiab=Heaijab=vfavjab=zeagbqaaiab=bfaqnaaBaaaleaacqWFObaAcqWF1bqDaeqaaaaaaaa@3430@

(*) In the system of differential equations, the sum (k_in_+k_er_) represents the length of the interval between infection (*sporozoite *inoculation) and the onset of infectivity (gametocyte maturation) in a vertebrate host. It may range from 15 to 19 days (for a mean value commonly used of 17 days), according to good, deteriorating or intermediate social and economic conditions (SEC) prevailing in the community [21]. The average immune and infectious periods (1/V and 1/γ, respectively) were estimated following the Martens assumptions [3]. (**) In simulation results the variable 'Prevalence' considers only the proportion of infectious individuals, expressed by the total number of infectious human hosts at a given time t scaled by the total human population at risk. Some authors define this endogenous variable as the overall malaria prevalence, therefore including both the infected and infectious stages.

**Table 5:** Exogenous variables considered in the infectious disease models

**Exogenous variable**	**Used variable**		**Depending on**	**Default value**
Human Biting Density	ma or HBD	[blood-meals/person/night]		Total mosquitoes captured (indoor and outdoor landing captures 06:00–09:00 pm)
Sporozoite Rate	s or SR	[dec]		0.07–0.12
Human susceptibility (efficiency with which an infective mosquito infects a susceptible human)	S_H	[dec]		1.00
Mosquito susceptibility (efficiency with which an infective human infects a susceptible mosquito)	S_V	[dec]		1.00
Proportion of infective female vectors (proportion of those anophelines with sporozoites in their salivary glands which are actually infective)	b	[dec]		0.01
Recovery rate in man	C1	[dec]		1.00
Transmission rate	f	[1/days]	f (Risk)	(G)

(G) The parameter *Transmission Rate f *is dependent on the average number of mosquitoes-bites on humans and measures the level of interaction between both populations. For a community in a low risk area of malaria *f *is set to 0.13; for an intermediate risk *f *is set to 0.17; and for a high-risk *f *is set to 0.25. This parameter can also be dependent on temperature (vectorial capacity), SEC (bed-nets and deforestation), and climate anomalies (El Niño event) [21].

**Table 6:** Endogenous variables considered in the infectious disease models

**Endogenous variable**	**Used variable**		**Depending on**	**Function**
Critical density	DCR	[mosquitoes/human]	S_H, S_V, a, p, n, C1	C1*−LN(p)S_H*S_V*a2*pn MathType@MTEF@5@5@+=feaafiart1ev1aaatCvAUfKttLearuWrP9MDH5MBPbIqV92AaeXatLxBI9gBaebbnrfifHhDYfgasaacH8akY=wiFfYdH8Gipec8Eeeu0xXdbba9frFj0=OqFfea0dXdd9vqai=hGuQ8kuc9pgc9s8qqaq=dirpe0xb9q8qiLsFr0=vr0=vr0dc8meaabaqaciaacaGaaeqabaqabeGadaaakeaaieqacqWFdbWqcqWFXaqmcqGGQaGkdaWcaaqaaiabgkHiTiab=Xeamjab=5eaonaabmGabaGae8hCaahacaGLOaGaayzkaaaabaGae83uamLaei4xa8Lae8hsaGKaeiOkaOIae83uamLaei4xa8Lae8NvayLaeiOkaOIae8xyae2aaWbaaSqabeaacqWFYaGmaaGccqGGQaGkcqWFWbaCdaahaaWcbeqaaiab=5gaUbaaaaaaaa@44C5@ [3]
Simulated density	DC	[mosquitoes/human]	VS, VI, VF, P_hu_	VS+VI+VFPhu MathType@MTEF@5@5@+=feaafiart1ev1aaatCvAUfKttLearuWrP9MDH5MBPbIqV92AaeXatLxBI9gBaebbnrfifHhDYfgasaacH8akY=wiFfYdH8Gipec8Eeeu0xXdbba9frFj0=OqFfea0dXdd9vqai=hGuQ8kuc9pgc9s8qqaq=dirpe0xb9q8qiLsFr0=vr0=vr0dc8meaabaqaciaacaGaaeqabaqabeGadaaakeaadaWcaaqaaGqabiab=zfawjab=nfatjabgUcaRiab=zfawjab=LeajjabgUcaRiab=zfawjab=zeagbqaaiab=bfaqnaaBaaaleaacqWFObaAcqWF1bqDaeqaaaaaaaa@3985@
Calibration parameter (VC)	K1		HBD, DCR, DC, D_0, D_1	
Entomological Inoculation Rate	EIR	[infected bites/human/night]	m, a, s, b	m*a*s*b
Basic Reproduction Rate	Z		VC, 1/V	VC*(1/V)
Interaction Susceptibility	IN_F	[dec]	R_IN	Decay function: 1.2 if R_IN = 0; 0.853 if R_IN = 2; 0.812 if R_IN = 5; and 0.802 if R_IN = 15
Vectorial Capacity	VC	[infected bites/human/night]	IN_F, K1, a, p, n	IN_F*K1*a2*pn−LN(p) MathType@MTEF@5@5@+=feaafiart1ev1aaatCvAUfKttLearuWrP9MDH5MBPbIqV92AaeXatLxBI9gBaebbnrfifHhDYfgasaacH8akY=wiFfYdH8Gipec8Eeeu0xXdbba9frFj0=OqFfea0dXdd9vqai=hGuQ8kuc9pgc9s8qqaq=dirpe0xb9q8qiLsFr0=vr0=vr0dc8meaabaqaciaacaGaaeqabaqabeGadaaakeaaieqacqWFjbqscqWFobGtcqGGFbWxcqWFgbGrcqGGQaGkcqWFlbWscqWFXaqmcqGGQaGkdaWcaaqaaiab=fgaHnaaCaaaleqabaGae8NmaidaaOGaeiOkaOIae8hCaa3aaWbaaSqabeaacqWFUbGBaaaakeaacqGHsislcqWFmbatcqWFobGtdaqadiqaaiab=bhaWbGaayjkaiaawMcaaaaaaaa@416A@ [3]

The function 〈(VS+VI+VF)⋅Rpo〉FI
 MathType@MTEF@5@5@+=feaafiart1ev1aaatCvAUfKttLearuWrP9MDH5MBPbIqV92AaeXatLxBI9gBaebbnrfifHhDYfgasaacH8akY=wiFfYdH8Gipec8Eeeu0xXdbba9frFj0=OqFfea0dXdd9vqai=hGuQ8kuc9pgc9s8qqaq=dirpe0xb9q8qiLsFr0=vr0=vr0dc8meaabaqaciaacaGaaeqabaqabeGadaaakeaadaaadeqaamaabmGabaacbeGae8NvayLae83uamLaey4kaSIae8NvayLae8xsaKKaey4kaSIae8NvayLae8NrayeacaGLOaGaayzkaaGaeyyXICTae8Nuai1aaSbaaSqaaiab=bhaWjab=9gaVbqabaaakiaawMYicaGLQmcadaWgaaWcbaGae8NrayKae8xsaKeabeaaaaa@4181@ represents the net oviposition that occur every FI pulse (see feeding interval in Table 2). That is, it is assumed that the interval between blood meals (i.e. the length of the gonotrophic cycle) is equivalent to the time interval between successive ovipositions. In the predator-prey interactions module, PD represents the predator population, a_S _the predation rate coefficient, b_p _the reproduction rate of predators per 1 prey eaten, and m_p _the predator mortality rate. In the breeding places availability model, f_L _and f_L_S _represent, respectively, the water availability and the level of desiccation (table functions FL_P and FL_P_S) affecting the egg laying in vector population into main module. To consider the preferences of ovipositing females for specific larval habitats, weighted factors of 0.40, 0.30, 0.10, 0.10, 0.05, and 0.05 were assumed for the breeding sites of 10, 20, 30, 40, 50, and 60 mm of capacity, respectively. Finally, the carrying capacity of the mosquito population was defined assuming a maximum vector-human ratio of 30:1.

### Study sites and demographic data

**Figure 2:**
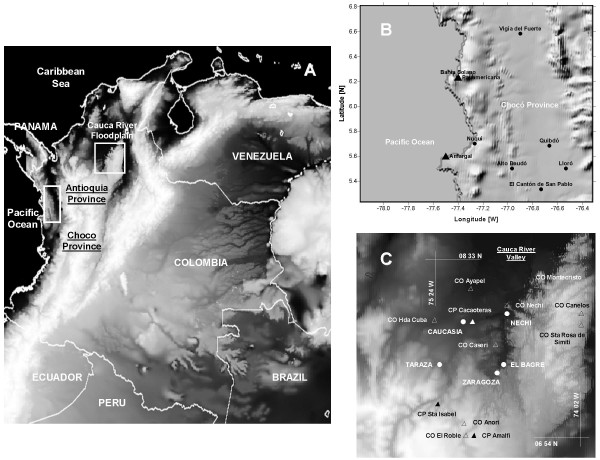
**Study sites**. Entomological, epidemiological and climatic data sets are being gathered for both the Nuqui and El Bagre regions (see figure A). The first study site is located on the Colombian Pacific coast (B); the El Bagre prone-region is located along the floodplains of the Cauca and Nechi Rivers, in north-western Colombia (C). The topography is represented using Digital Elevation Models for a 30"-arc spatial resolution. The circles represent the location of principal towns. The triangles represent the location of nearby weather stations (ordinary weather stations CO and principal weather stations CP).

Previous diagnostic studies of the relationship between climate and malaria in Colombia focused on a nationwide level at yearly timescales [6-8], and on regional and local levels at inter-annual, annual and seasonal timescales [5]. Previous modelling efforts by the research group were applied to understand malaria transmission in an endemic site on the Pacific coast of Colombia (the Nuqui Region) [30-32]. For this application, the simulation period of the mentioned study site was extended and a new specific area located in the Department of Antioquia, in north-western Colombia (Figure 2) was chosen. This area, or the so-called 'El Bagre' Region, is located in the 'Bajo Cauca' Programmatic Area along the floodplains of the Cauca and Nechi Rivers (Figure 2C). The current total populations in Nuqui and El Bagre localities reach, according to demographic census, 5,324 and 65,342 individuals, respectively, with 2,565 (48,2%) and 24,470 inhabitants (37,4%) living in rural areas. Demographic analysis indicated that the growth rates for these municipalities reached -0,72% (decrease) and 3,09% (increase), respectively. In the study sites, several climatic, epidemiological and entomological data sets were gathered, and malaria incidences were simulated under conditions of intense transmission on a daily timescale.

### Climate data, missing periods and homogeneity analysis

**Figure 3:**
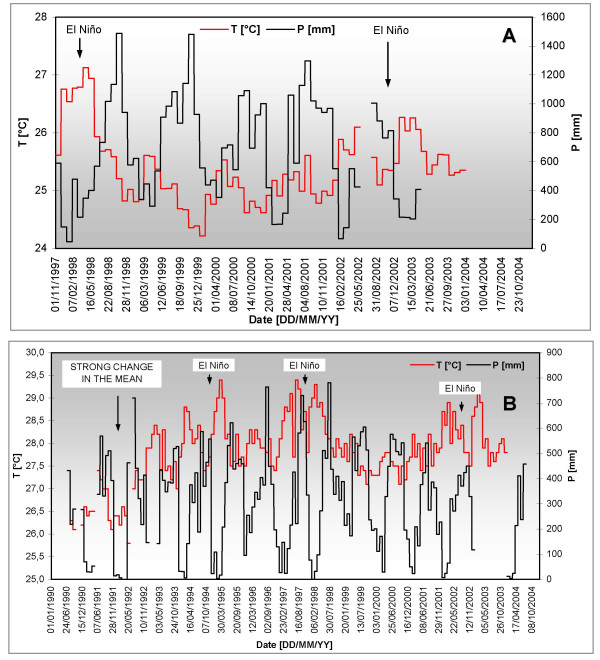
**Historical hydroclimatic time series**. Mean monthly temperatures (T [°C]) along with the total monthly precipitation records (P [mm]) recorded in Nuqui (A) and El Bagre (B). The warm event (El Niño) comprised 4 periods: 1991–1992, 1994–1995, 1997–1998, and 2002–2003. The cold event (La Niña, not shown) comprised 2 periods: 1995–1996 and 1998–1999.

Mean daily temperature values [°C], mean daily relative humidity values [%], and total daily precipitation records [mm] were registered at two nearby weather stations: Amargal at 05°36'N, 77°30'W and altitude 30 m, in the Nuqui Region, and Caseri at 07°49'N, 74°55'W and 400 m, in the El Bagre Region. Data pertaining to the periods comprising from November 1^st^, 1997 through December 31^st^, 2004 (2,618 days or 86 months) and from January 1^st^, 1990 through December 31^st^, 2004 (5,479 days or 180 months) were obtained for the Nuqui and El Bagre endemic areas, respectively. Figure 3 illustrates the time series of these historical hydroclimatic records on a monthly timescale. Detailed information of available and missing periods for the weather stations is presented in Table 7.

**Table 7:** Description of the climate data

Endemic region	Time period	Historical time series	Daily timescale		Monthly timescale	
			
			Total	Missing	Total	Missing
Nuqui	Nov/01/1997 – Dec/31/2004	Mean daily temperatures	2,618 days	459	86 months	14
		Total daily rainfall		672		22
		Mean daily relative humidity values		462		14
El Bagre	Jan/01/1990 – Dec/31/2004	Mean daily temperatures	5,479 days	1,234	180 months	23
		Total daily rainfall		1,018		30
		Mean daily relative humidity values		1,561		27

As mentioned above, homogeneity analyses were conducted to detect changes in the mean, variance, as well as significant trends in the climatic data sets. To detect the point of change in the mean and/or the variance in each single hydrological time series the Bayesian Analysis (BA) and the Abbe' Criterion for Homogeneity Test were used, both at a 0.05 significance level. The BA allowed determining the mean and the mode of the strongest point of change (NC point) and the mean of the total amount of change. The Abbe test allowed determining whether the hypothesis of homogeneity of each historical time series could be accepted or rejected.

To detect changes in the variance of each single hydrological time series (using the point of change estimated by the BA), the Simple F Test, the Simple F Test with corrections of dependence, the Modified F Test using both Chi-square and F distributions, the Ansari-Bradley Test, the Bartlett Test, and the Levene Test were conducted, all at a 0.05 significance level.

To detect changes in the mean of each single hydrological time series (using the point of change estimated by the BA), the Mann-Whitney/Wilcoxon Rank Sum Test, the Simple T Test assuming change and no change in the variance, the Modified T Test assuming change and no change in the variance, the Simple T Test with corrections of dependence assuming change and no change in the variance, and the Kruskal-Wallis Test were conducted, all at a 0.05 significance level.

To detect trends in the historical time series (free of seasonality) the T Test for the detection of linear trends, the Hotelling-Pabst Test, the Man-Kendall Test, and the Sen Test were conducted, all at a 0.05 significance level.

Finally, to detect trends in the seasonal time series the T Test for the detection of linear trends, the Hotelling-Pabst Test, the Seasonal Kendall Test, and the Seasonal Homogeneity of Trends Test were conducted, all at a 0.05 significance level.

All these hypotheses tests were run using the ASH (Analysis of Historical Time Series) Software developed by J.D. Salas and R.A. Smith at the Hydrology and Water Resources Program, Colorado State University, and recently modified at the Water Resources Graduate Program, National University of Colombia at Medellin.

The hypotheses tests for detecting non-homogeneities in time series recorded in the Nuqui Region showed that the historical records are homogeneous for the entire available period (results not shown). Both time series of mean monthly temperatures and mean monthly relative humidity values registered in the El Bagre region exhibit, on the other hand, strong non-stationarities in the mean and variance in May, 1992 (Figure 3B). As the shift showed not to be climate-induced, it was considered that only historical records can be assumed homogenous from July 1^st^, 1992 onwards. Noteworthy, mean monthly temperatures observed in both regions showed strong increases in mean annual temperatures (approximately +1.0 – +1.5°C) during the onset of the El Niño warm event, which comprised the periods 1994–1995, 1997–1998 and 2002–2003 (Figure 3).

Finally, all daily missing records for both temperature and relative humidity were substituted by an estimated value based on observed monthly records. Missing daily precipitation records were reconstructed assuming no rain on each day.

### Epidemiological data, statistical and correlation analyses

**Figure 4:**
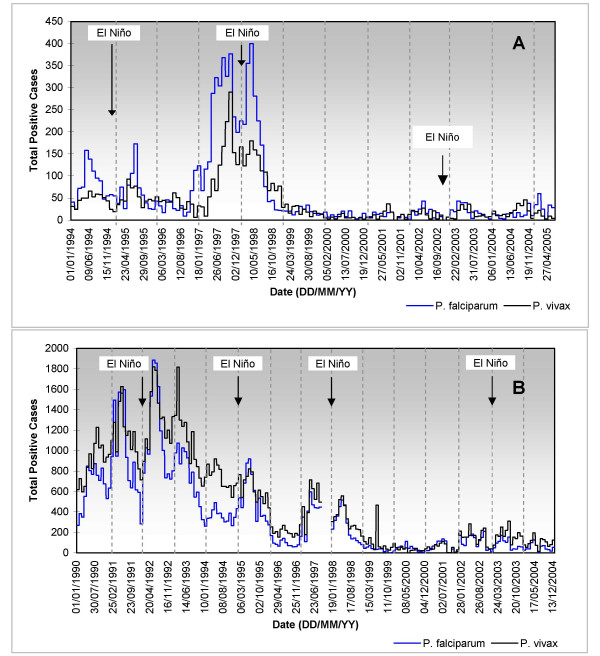
**Historical epidemiological time series**. Total positive cases of *P. falciparum *and *P. vivax *malaria recorded in Nuqui (A) and El Bagre (B) during the periods from January 1994 to June 2005 (monthly timescale) and from January 1990 to December 2004 (1^st ^EP 1990-13^th ^EP 2004), respectively. The Antioquia Health Service and the National Institute of Health provided malaria data for El Bagre, for the periods 1^st ^EP 1990-13^th ^EP 2000 and 1^st ^EP 2000-13^th ^EP 2004, respectively. In this area, missing data span the period from September 8^th^, 1997 to December 28^th^,1997 (10^th ^EP 1997-13^th ^EP 1997).

Unfortunately, in Colombia several health agencies have been in charge of monitoring malaria disease. The Malaria Eradication Service, a NGO operating on a nationwide level and working for the Ministry of Health, collected data on positive malaria cases from 1984 through 1993. Regional Health Services, public agencies operating at the regional level, were in charge of storing and processing information for each municipality in each Department from 1993 through 1998. In this case, regional authorities split the calendar year, for operational purposes, into 13 epidemiological periods (EP) of 4 weeks each. Roughly speaking, it can be assumed that the first EP corresponds to the month of January and the thirteenth EP corresponds to the month of December. More recently the National Public Health Surveillance System, an operative Department of the Colombian National Institute of Health, has been collecting data on positive malaria cases on the local level for each epidemiological week from 1998 onwards. Figure 4 illustrates the time series of these historical epidemiological records on monthly or per EP timescales.

Even though historical time series seem to be homogeneous in the Nuqui region, the continuous changes in the responsibilities of the local authorities have lead, on the contrary, to several non-homogeneities in epidemiological time series of the El Bagre region (Figure 4B). In this case, strong changes in the mean were detected in the epidemiological records. Although the errors or omissions appear to be important in such historical time series, datasets were only checked for accuracy, logic and range of values.

In the Nuquí region, monthly epidemiological data comprises the continuous period of January, 1994 through June, 2005 (138 months or 4,199 days). The Antioquia Health Service and the National Institute of Health provided regional malaria data for the El Bagre region, for the period spanning from January 1^st^, 1990 through December 31^st^, 2004 (1st EP 1990-13th EP 2004; 180 months or 5,479 days).

These data sets were collected according to the type of infection (*P. falciparum*, *P. vivax*, and mixed malaria) and age groups (<1, 1–4, 5–14, 15–44, 45–59, and >60 years), and were used to develop different types of statistical analyses for understanding the linear and no-linear degree of correlation between climatic variables and malaria transmission indexes. Statistical and correlation analyses were conducted using both the Microsoft Excel Data Analysis Tool and the SPSS version 11.5. The central tendency and variability of all these data sets were examined using descriptive statistics. Finally, the relationship between climatic variables and malaria cases (or incidences) was explored using linear regression and cross correlation with various time lags.

Time series regarding epidemiological data showed to be statistically associated with ambient temperatures and precipitation records during the continuous and homogenous climatic data sets, which comprised the periods from November, 1997 to May, 2002 and from May, 1992 to November, 2000 for the Nuqui and El Bagre regions, respectively. Historical time series for both positive *P. vivax *and *P. falciparum *malaria incidences registered in the first locality exhibited a significant degree of correlation with mean monthly ambient temperature time series, for a 1-month time lag (see Table 8). Correlation coefficients reached, respectively, positive values of 0.833 and 0.827 (P > 0.95). Time series also exhibited a significant degree of correlation with total monthly precipitation time series for a 3-months time lag. In this case, correlation coefficients reached, respectively, negative values of -0.400 and -0.447 (P > 0.95). *P. vivax *and *P. falciparum *malaria incidences observed in the El Bagre region (per epidemiological period) were positively correlated with mean temperatures (R = 0.323 and R = 0.487, respectively), for a time lag of 1 EP (Table 8). Analyses show that precipitation time series has a lag of 4 EP and negative correlation coefficients of about -0.435 and -0.496 (P > 0.95).

**Table 8:** Correlation analysis

Nuqui region					El Bagre region				
Lag (month)	IVM (T)	IVM (P)	IFM (T)	IFM (P)	Lag (EP)	IVEP (T)	IVEP (P)	IFEP (T)	IFEP (P)

0	0.772	-0.250	0.810	-0.332	0	0.315	-0.155	0.385	-0.067
1	0.833	-0.293	0.827	-0.384	1	0.323	-0.317	0.487	-0.315
2	0.818	-0.301	0.788	-0.409	2	0.312	-0.349	0.466	-0.436
3	0.832	-0.400	0.733	-0.447	3	0.342	-0.416	0.472	-0.491
4	0.790	-0.441	0.608	-0.413	4	0.280	-0.435	0.398	-0.496

Correlation coefficients for several time lags between incidences of both *P. vivax *(IVM or IVEP) and *P. falciparum *(IFM or IFEP) malaria, and mean monthly (per EP) ambient temperature (T), as well as total monthly (per EP) precipitation records (P).

Finally, correlation analyses show that epidemiological time series seem to be strongly associated with mean temperatures, particularly during the onset of the El Niño warm event, as shown in Figure 4. Noteworthy, similar increments in the number of malaria cases during the El Niño event have been found throughout Colombia [5,6,33,34].

**Figure 5:**
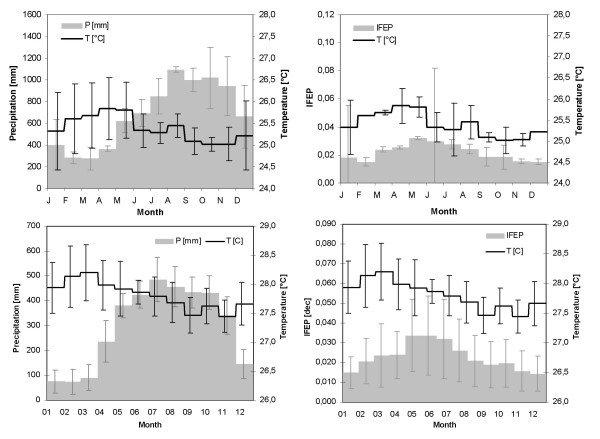
**Annual cycles of rainfall, temperature and malaria incidences under 'average' conditions**. Intra-annual cycles of total monthly rainfall and mean monthly temperature patterns observed in Nuqui (top left) and El Bagre (bottom left) under 'average' conditions during the periods 1997–2003 and 1992–2004, respectively. Annual cycles of average monthly (per EP) malaria incidence in Nuqui (top right) and El Bagre (bottom right) along with mean monthly temperatures. Error bars indicate the confidence interval for a 0.05 significance level.

Figure 5 depicts the intra-annual cycles of rainfall and temperature patterns observed in the Nuqui and El Bagre regions under 'average' conditions, along with the average monthly (or per epidemiological period) *P. falciparum *malaria incidences. Analysis of total monthly precipitation records for the period 1997–2003 indicates that under 'average' conditions the rainfall patterns in the Nuqui region exhibit an intra-annual cycle with one peak, corresponding to the rainy season, which commonly occurs during the September-October-November trimester. In the El Bagre region the rainfall patterns, analysed for the period 1992–2004, also exhibit an intra-annual cycle with one peak, although the long rainy season typically occurs during July-August-September. Analysis of the mean monthly temperatures observed in the Nuqui region for the period 1997–2003 indicates that under 'average' conditions a highly warm season typically falls in February-March-April, and a period of low temperatures normally occurs in September-October-November. These patterns are also observed in the El Bagre region during the period 1992–2004. The average monthly incidence of *P. falciparum *malaria for the Nuqui region during the period 1997–2003 shows that, under 'average' conditions, malaria incidence reaches a peak in transmission around the months of May and June, following the period of high temperatures and with the onset of the rainy season. These patterns of *P. falciparum *malaria were also observed in the El Bagre region during the period 1992–2004.

**Figure 6:**
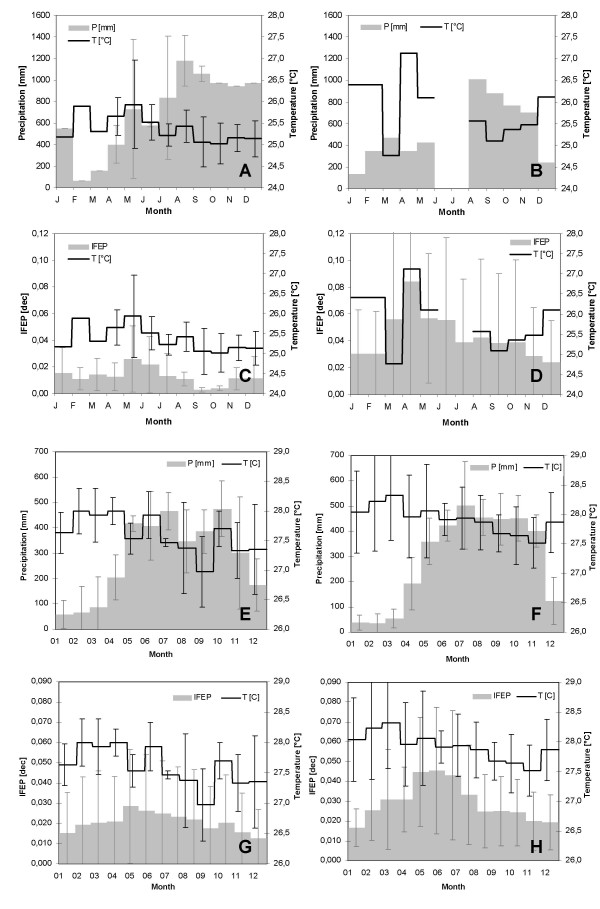
**Annual cycles of rainfall, temperature and malaria incidences under 'normal' and El Niño conditions**. Intra-annual cycles of total monthly rainfall and mean monthly temperature patterns observed in Nuqui under 'normal' (A) and El Niño (B) conditions during the period 1997–2003. Intra-annual cycles of average monthly malaria incidence and mean monthly temperatures recorded in this malaria prone-region under 'normal' (C) and El Niño (D) conditions. Intra-annual cycles of total monthly rainfall and mean monthly temperature patterns observed in El Bagre under 'normal' (E) and El Niño (F) conditions during the period 1992–2004. Intra-annual cycles of average malaria incidence per epidemiological period and mean monthly temperatures recorded in El Bagre under 'normal' (G) and El Niño (H) conditions. Error bars indicate the confidence interval for a 0.05 significance level.

Figure 6 depicts the aforementioned annual cycles observed in the Nuqui and El Bagre regions, but under 'normal' and El Niño conditions. Analysis of the total monthly precipitation records indicates that, under the extreme weather event, rainfall patterns in the Nuqui region exhibit a significant decrease during the months of December and January. Monthly rainfall records fall to values of about 200 mm compared to the average observed records, which under 'normal' conditions reach almost 1,000 and 550 mm, respectively. In the El Bagre region, the El Niño warm event almost causes severe drought during the months of January-February-March. Monthly precipitation records, which under 'normal' conditions reach values of about 100 to 200 mm, fall drastically to values below 50 mm. It is worth mentioning that the period of long rainy seasons seems not to be significantly altered in this locality. Analysis of temperature patterns in the Nuqui region shows that, under El Niño conditions, the mean annual temperature increases by about 0.7°C and the mean monthly temperatures during the months of December and January reach 26.5°C, compared to observed values under 'normal' conditions of about 25.0°C. These patterns are also observed in the El Bagre region, although the increase in mean annual temperature reaches 0.5°C and the months affected are January-February-March. In several instances monthly records reach, in this case, values of 29°C compared to average values observed under 'normal' conditions of about 28°C. Finally, and as mentioned above, the El Niño event seems to intensify the annual cycle of malaria cases in both regions as a consequence of the anomalies in the normal patterns of temperature and precipitation. The average monthly incidence of *P. falciparum *malaria in the Nuqui region shows that, under 'normal' conditions, malaria incidence reaches a peak in transmission of about 2% of the total population at risk during the months of May and June. During the El Niño events, the annual cycle of malaria incidence remains with one peak, around the month of April (the timing almost remains unaltered), although the number of cases (amplitude of the malaria outbreak) increases significantly. Based on the available epidemiological period, it can be argued that malaria incidence in the Nuqui region during the extreme weather event can increase to an average value of about 8% of the total population at risk, with maximum values reaching 12%. In the El Bagre region malaria incidence reaches a peak in transmission under 'normal' conditions of 3 to 5% of the total population at risk, and also during the months of May and June. During the El Niño events, a sharp rise in the number of cases was commonly observed in the locality, which led to malaria incidences affecting between 5 to 8% of the total population at risk (Figure 6).

### Base scenarios

Roughly speaking, it is generally accepted that there are two scenarios during which malaria transmission could be possible in Colombian endemic-regions: (1) High levels of disease transmission could be expected during periods of 'excellent' conditions following long rainy seasons, when mosquito densities are generally high and temperatures are not low enough to inhibit a successful parasite development within the mosquito host. This scenario is primarily controlled by vector density and suggests that temperature and precipitation variables might have synergistic effects on malaria transmission. Temperature affects those entomological variables relevant to the biology of the mosquito host mainly during its aquatic stages; precipitation controls the availability (and, in turn productivity) of adequate breeding sites. This scenario requires linking and simulating the dynamics of vector ecology and malaria transmission in adult mosquitoes and human hosts. (2) High levels of disease transmission could be also expected during periods of 'good' conditions following warm dry seasons, periods when vector densities tend to be extraordinarily low. Although this scenario is 'good' in terms of temperature for the development of parasites in the mosquito host, it seems not to be particularly favourable for disease transmission in terms of low vector densities. Under this scenario, transmission is primarily controlled by those entomological variables (sporogonic cycle and feeding interval) that are strongly affected by temperature. High temperatures could lead to a shortening of the duration of both the extrinsic incubation period and the gonotrophic cycle. The simulation of the dynamics of malaria transmission could be then simplified by estimating a constant vector density (or a smooth seasonal fluctuation of vector density) and separately simulating the disease transmission in human hosts.

As the analysis of annual cycles of rainfall, temperature and malaria incidence patterns shows peaks in malaria transmission following the periods of high temperatures (and when rainy seasons starts to occur), as shown in Figures 5 and 6, it was proposed the second epidemiological scenario discussed above for the Nuqui and El Bagre regions. Hence, malaria transmission was simulated assuming the Vectorial Capacity infectious disease model for specific constant mosquito densities, which were estimated through the analysis of the availability of larval habitats in the selected localities.

**Figure 7:**
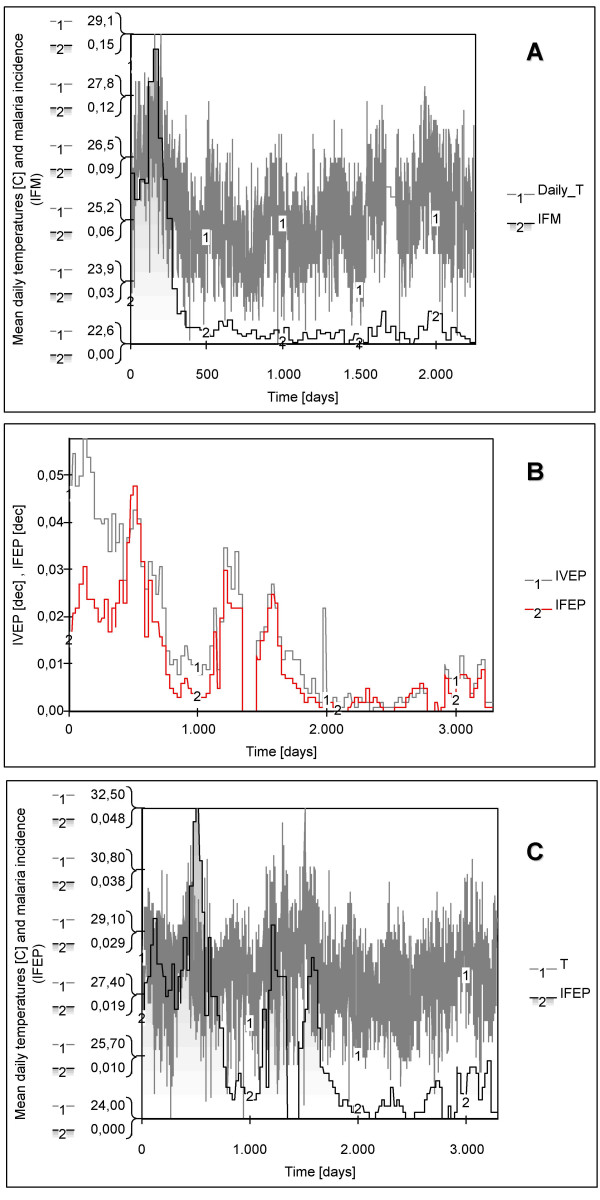
**Time series of climatic and epidemiological data observed during the simulation periods**. (A) Monthly *Plasmodium falciparum *malaria incidence (IFM: total positive reported cases scaled by the total population at risk) for the Nuqui region along with mean daily temperatures (Daily_T) recorded during the period from November 1^st^, 1997 to December 31^st^, 2003 (2,252-day simulation period). (B) *P. falciparum *(IFEP) and *P. vivax *(IVEP) malaria incidences recorded in El Bagre, every epidemiological period, during the period from January 3^rd^, 1994 to December 28^th^, 2002 (1^st ^EP 1994-13^th ^EP 2002). In this case, the simulation exercise comprises a 3,282-day simulation period. (C) *Plasmodium falciparum *malaria incidence (IFEP, gray bars) for El Bagre along with mean daily temperatures (T, solid line). Notice in the graphs the strong increasing and decreasing trends. The plots indicate that the increase in the number of *P. falciparum *malaria cases might be associated with increases in air temperatures.

In the Nuqui region monthly values of *P. falciparum *malaria incidence were modelled for the period of November 1^st^, 1997 to December 31^st^, 2003. This period corresponds to a simulation exercise of about 2,252 days or 74 months or, approximately, 6 years (Figure 7A). The initial total human population at risk was assumed to be equal to 2,835 people, the population of the locality living in rural areas at the end of 1997. No human migration was assumed to have taken place during the simulation period. The initial number of total infectious individuals HUF(0) was assumed to be equal to 377 people, equivalent to the total number of positive cases reported during October, 1997. The initial number of total infected individuals HUI(0) was assumed to be equal to 234 people, equivalent to the total number of positive cases reported during November, 1997. The initial number of total immune individuals HUM(0) was assumed to be equal to 326 people, equivalent to the total number of positive cases reported during September, 1997. In this case it was proposed that individuals remained in the infectious stage during a short period of time (greater, but close to the duration of treatment) and experienced a re-exposure to malaria parasites. Finally, the initial number of total susceptible individuals HUS(0) was estimated to be the difference between the total population at risk and the total initial number of people in the infectious, infective and immune stages.

Based on the continuous and homogeneous periods of climatic and epidemiological data sets available in the El Bagre region, the observed *P. falciparum *malaria incidence in the study site was modelled for the period from January 3^rd^, 1994 to December 28^th^, 2002 (first epidemiological period 1994 – 13th EP 2002). This period corresponds to a simulation exercise of about 3,282 days or 108 months, or 9 years (Figures 7B and 7C). The initial total human population at risk was assumed to be equal to 15,862 people, the population of rural areas at the end of 1994. The initial number of total infectious, infected and immune individuals was assumed to be equal to HUF(0) = 326, HUI(0) = 261, and HUM(0) = 453 people, equivalent to the total number of positive cases reported during the 13^th ^EP 1993, 1^st ^EP 1994, and 12^th ^EP 1993, respectively. Accordingly, the total number of susceptible individuals HUS(0) was assumed to be equal to 14,822 individuals. In the endemic area, malaria incidence was simulated assuming temperature values equal to either mean daily indoor temperatures or a constant value of 27.7°C, equivalent to the mean annual temperature of this malaria prone-region.

The system of coupled differential equations was solved by using a fourth order Runge-Kutta numerical algorithm for a 1-day time step at both study sites. Values of exogenous variables are shown in Tables 1, 3 and 5.

### Preliminary analysis of instability cases

**Figure 8:**
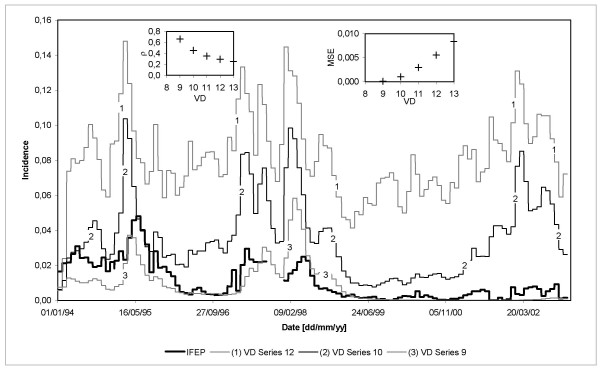
**Time series of model results for several vector densities**. ρ and MSE represent the correlation coefficient and the mean square error, respectively, obtained by the comparison of observed *P. falciparum *malaria incidence rates and simulated disease prevalence at EP time resolution.

Based upon the aforementioned scenario and following the experimentation-validation processes, small changes in some exogenous variables were assumed in the analysis of instability cases. Simulations included changes in vector density (Figure 8), Human Blood Index, number of degree-days required for parasite development, human average infected period, and Primary *Exo-erythrocytic Schizogony *time delay. Analysis of instability cases were only conducted for the 3,282-day simulation period considered for the El Bagre region.

### Sensitivity to initial conditions

**Figure 9:**
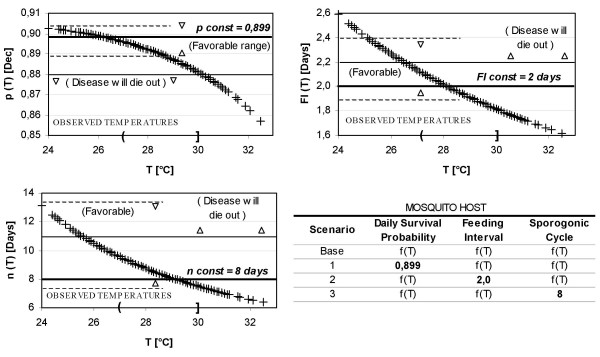
**Parameters and scenarios considered for sensitivity analysis**. Estimated values of daily survival probability of the mosquito host (p [dec]), feeding interval (FI [days]), and sporogonic period (n [days]) for the mean daily temperatures recorded in El Bagre region.

Several parameters that might cause sensitivity to initial conditions in simulation results were also investigated (Figure 9). Only three scenarios were analysed in these preliminary simulations: (1) Scenario 1 set the daily survival probability of the mosquito host to a constant value and considered both the gonotrophic and sporogonic cycles to be functions of mean daily temperatures. (2) Scenario 2 set the feeding interval of the mosquito host to a constant value and considered both the daily survival probability and the sporogonic cycle to be functions of mean daily temperatures. Finally, (3) scenario 3 set the sporogonic cycle to a constant value and considered both the daily survival probability and the feeding interval to be functions of mean daily temperatures. Analyses were only conducted for the El Bagre region and the 3282-day simulation period.

**Figure 10:**
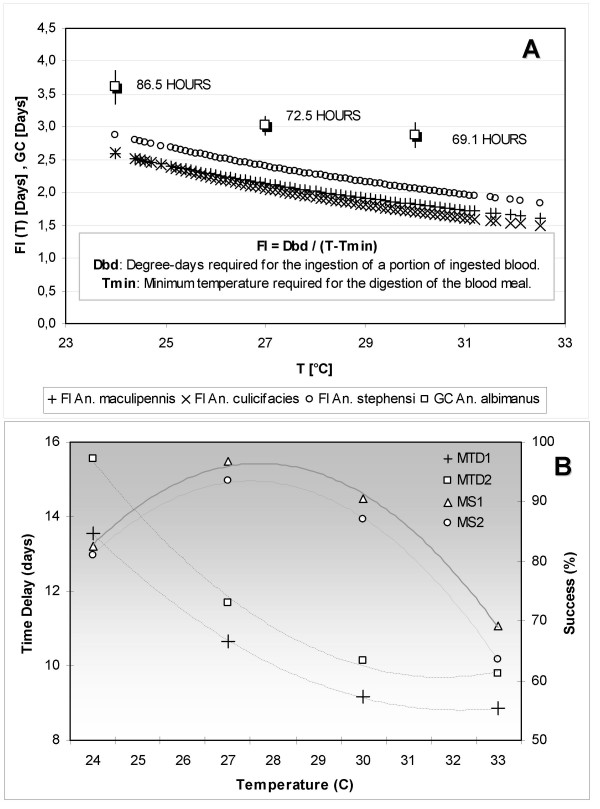
**Analysis of entomological variables under controlled laboratory conditions**. (A) Feeding intervals and observed gonotrophic cycle. The frequency of feeding was estimated from literature for *An. maculipennis*, *An. culicifacies *and *An. stephensi*: 36.5°C-days and 9.9°C of threshold temperature, 29.7°C-days and 12.6°C of threshold temperature, and 43.4°C-days and 8.9°C of threshold temperature [37], respectively. The duration of the gonotrophic cycle was estimated under controlled laboratory conditions for *An. albimanus *at several temperatures (see also mean value and 95% confidence intervals). (B) Average periods of time spent in the compartments L and PU, along with mean success for both larvae and pupae for *An. albimanus*. These variables are needed to estimate the proportion of larvae and pupae becoming non-viable. MTD1 and MTD2 represent the mean time delays from larva-1st instar to pupa and adult, respectively. MS1 and MS2 represent the mean successes from larva-1st instar to pupa and adult, respectively.

To simulate the base scenario and conduct the analysis of instability cases and sensitivity to initial conditions, the values reported in literature for the daily survival probability (longevity), the sporogonic period and the feeding interval were compared with either field-based approximations or values estimated under controlled laboratory conditions (Figure 10). The daily survival probability was contrasted with the nulliparous-to-parous ratio estimated from collections of adult female mosquitoes captured through the Human Landing method [2]. Field campaigns were conducted during at least three consecutive evenings per month, both indoors and outdoors around houses located at the study sites. Primary vectors of malaria transmission captured in these areas were *Anopheles albimanus *on the Colombian Pacific Coast, and *Anopheles darlingi *on the floodplains of Cauca and Nechi rivers. The reported feeding interval was compared to the duration of gonotrophic cycle of *An. albimanus *estimated under controlled laboratory conditions, as shown in Figure 10. The research group is currently estimating the duration of the sporogonic period.

### Climate scenarios

Four changing climate scenarios were simulated for the El Bagre region assuming increases in mean daily temperatures for the entire simulation period from January 3^rd^, 1994 to December 28^th^, 2002. Based on the Special Report on Emission Scenarios and the predicted Colombian Climate Change Scenarios for year 2050 (particularly for the study site), increases of 1.0°C, 1.6°C, 1.9°C, and 2.8°C in mean daily ambient temperatures were assumed. Although it does not simulate a future scenario *per se*, it represents the possible dynamics of malaria transmission of an endemic area in which a population with similar characteristics of the El Bagre region lives. Hence, simulation of a changing climate scenario will only allow for the realization of a strong non-linearity involved in the mathematical model.

## Results

### Vector densities

During a 1,800-day period assumed for the El Bagre region (January 3rd 1994 – December 7th 1998) mean daily temperatures ranged from 24.0 to 32.5°C, and at least 83% of the observed values were in the interval (27-30°C]. Within these temperature ranges, the critical density necessary to maintain parasite transmission ranged from 16.9 to 6.6, and from 8.9 to 6.7 mosquitoes per human host, respectively. The assumed simulation period also showed five wet seasons, which are generally associated with peaks in vector densities, and four dry seasons, which commonly lead to low levels in this entomological variable. Simulation results of the dynamics of mosquito ecology showed high vector densities of about 15, 17, 12, 17, and 12 mosquitoes per human host during these wet seasons, and low values of about 3, 5, 4, and 3 vectors during dry periods in this study site.

Similar analyses were conducted for the Nuqui region although results are not discussed here. However, it is worth mentioning that during the simulation period of November 1^st^, 1997 to December 31^st^, 2003 (2,252 days), mean daily temperatures in this area ranged from 22.6 to 29.1°C, and the critical density necessary to maintain parasite transmission reached values ranging from 7.5 to 28.6 mosquitoes per human host.

The combined analyses of availability of breeding sites, predator-prey interactions, critical densities for malaria transmission, and dynamics of vector ecology, showed that constant vector densities, ranging from 7 to 12 mosquitoes per human host, might be considered for simulating the dynamics of malaria transmission and conducting the analysis of instability cases in the El Bagre region. Similar simulation exercises carried out for the Nuqui region showed constant vector densities ranging from 10 to 16 mosquitoes per individual.

Finally, simulation results also showed average vector densities of about 11, 10, and 9 mosquitoes per human host for the periods January 3^rd^, 1994 to March 12^th^, 1996 (time steps 1–800), March 13^th^, 1996 to November 2^nd^, 1997 (801–1,400), and November 3^rd^, 1997 to December 7^th^, 1998 (1,401–1,800), if a crude seasonality of vector density is desired for simulating malaria transmission in the El Bagre region.

### Base scenarios

**Figure 11:**
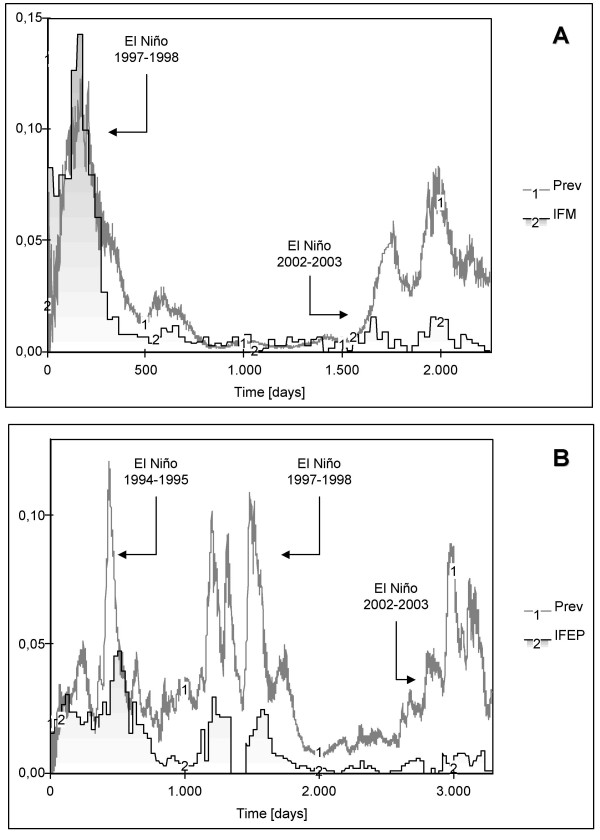
**Simulation results for the assumed basescenarios**. (A) IFM denotes the monthly *P. falciparum *malaria incidence (gray bars) observed in Nuqui during the period November 1st 1997-December 31st 2003 (2,252-day simulation period). (B) IFEP denotes the *P. falciparum *malaria incidence (gray bars) observed per EP in El Bagre during the period January 3rd 1994 – December 28th 2002 (3,282-day simulation period). Variables Prev represent the *daily *malaria prevalence rates (solid lines) obtained by simulation using daily temperatures and the 'Vectorial Capacity' infectious disease model for constant vector densities.

Constant mosquito densities of 14 and 10 mosquitoes per human host were initially assumed for the Nuqui and El Bagre regions, respectively. Observed malaria incidence rates and simulated disease prevalence values were compared at a monthly (or per epidemiological period) time resolution by estimating both the correlation coefficient (R) and the mean square error (MSE). Simulation results for the assumed base scenarios exhibited values of R of about 0.727 and 0.455 (P > 0.95), and MSE of 0.0006 and 0.0010, for the Nuqui and El Bagre regions, respectively (see Figure 11 for a daily timescale in simulated incidence). In the first endemic area, modelling results show that seasonality, observed prevalence and time of occurrence of recorded malaria outbreaks were 'well' simulated. Although the observed sharp rise in malaria incidence during the El Niño 1997–1998 was well represented (time steps 1–501), the model shows that outbreaks recorded during the period 1,601–2,252 were overestimated (Figure 11A). In the El Bagre region modelling results for mean daily temperatures show that seasonal fluctuations of disease transmission and incidence rates were 'well' represented, except for the time steps 2,801–3,282 when prevalence was overestimated (Figure 11B). Nevertheless, simulated prevalence exhibits peaks in transmission two and three epidemiological periods before recorded outbreaks. That is, prevalence rates and observed incidences were delayed in this study site (Figure 11B). For a constant value of 27.7°C, equivalent to the mean annual temperature of the El Bagre region, modelling results (not included) show that prevalence oscillates during the first 100 days of the simulation period, increases almost linearly from 0.8% to 1.4% during the time steps 100–1,000, and reaches an equilibrium point of 1.43% of total population at risk. Modelling results in both endemic areas showed that, as expected, temperature became the most relevant climatic variable strongly affecting those entomological variables considered relevant for disease transmission and incidence.

Under the base scenario, the Vectorial Capacity (VC) and the Basic Reproduction Rate (R_o_) estimated for El Bagre showed values ranging from 0.6 to 1.8 potential infective bites per day, and from 0.5 to 1.8 arising secondary cases, respectively, for mean daily outdoor temperatures and the assumed vector density. For the interval 27–30°C, VC ranged from 1.0 up to 1.8 daily potential infective bites. In Nuqui, VC and R_o _reached values in the ranges 0.5–1.9 and 0.5–2.1, respectively.

The estimated R_o _for the El Bagre region showed a broad range of values although all of them remained at lower levels. For temperatures below 27°C, R_o _showed values below the transmission threshold (R_o _= 1) meaning that the disease cannot develop in this community. For temperatures above 27°C, most of R_o _values ranged between 1.0 and 2.0, suggesting that the disease can develop in the population at risk but at low endemic levels. In Nuqui, R_o _reached values below the transmission threshold for temperatures below 25°C. For temperatures above this threshold, R_o _values ranged linearly between 1.0 and 2.0.

### Instability cases

In El Bagre, simulation results for changes in vector density show low correlation coefficients (less than 0.351) and high mean square errors (above 0.0030) for densities greater than 11 mosquitoes per human host (Figure 8). For instance, if vector density is set to a constant value of 12 mosquitoes per host, the model significantly overestimates observed malaria incidence (R = 0.293 and MSE = 0.0055; see solid line 1). For values of this entomological variable lower than 7–8 (below the critical density threshold necessary to maintain parasite transmission), the model shows, as expected, that malaria disease recedes and dies out due to the low values of the Basic Reproduction Rate. If vector density is set to 9 mosquitoes per human host, the model represents (R = 0.662 and MSE = 0.0001, see solid line 3) the observed malaria incidence but mainly for the period from January 1994 to July 1999. If vector density is set to 10 mosquitoes per human host (base scenario, see solid line 2), the correlation coefficient drops to 0.455 and the mean square error reaches 0.0010. In this case, the model adequately represents the malaria incidence for the period from January 1994 to November 2000. Modelling results suggest that a density of about 9–10 mosquitoes per human host are required for the development of malaria in the community at risk of the El Bagre region.

Simulation results (not included) for changes in Human Blood Index (HBI) show correlation coefficients and mean square errors reaching 0.455–0.395 and 0.0010–0.0019, respectively, for values of HBI in the range 0.41–0.42. For values above 0.44, the model considerably overestimates observed malaria incidences. In this case, simulation results show low correlation coefficients (less than 0.318) and high mean square errors (greater than 0.0042). If HBI is set to 0.38, the model is not able to reproduce either the observed malaria incidence or the recorded outbreaks. Modelling results suggest that a Human Blood Index of about 0.40–0.41 is required for the development of malaria in the community at risk. If this exogenous variable is set to these values, the model adequately represents malaria incidence but mainly for the period from January 1994 to November 2000.

Simulation results (not included) for changes in the number of Degree Days for Parasite Development (DD) in the mosquito host show high correlation coefficients (0.455–0.529) and low mean square errors (0.0010–0.0005) for values of DD in the range 111–115°C-days. If DD is set to 111°C-days, the Extrinsic Incubation Period (EIP) or sporogonic cycle decreases from 13.9 days at 24°C to 7.0 days at 32°C. If this exogenous variable is set to 115°C-days, the EIP is shortened from 14.4 days at 24°C to 7.2 days at 32°C. If DD is set to 120°C-days (EIP ranging between 15 and 7.5 days) the correlation coefficient increases to 0.675 and the mean square error decreases to 0.0001. If DD is set to 105°C-days (EIP ranging between13.1 and 6.6 days), R drops to 0.383 and MSE increases to 0.0021. In this case, the mathematical model considerably overestimates observed malaria incidences.

Simulation results (not included) for changes in the Human Average Infected Period (ν) show correlation coefficients and mean square errors reaching 0.455–0.315 and 0.0010–0.0059, respectively, for values of ν in the range 0.95–1.10 years. If ν is set to 1.50 years, the correlation coefficient drops to 0.199 and the mean square error significantly increases to 0.0403. In this case, the model overestimates the recorded malaria incidence. If this exogenous variable is set to 0.80 years, the model shows that malaria disease recedes and dies out due to low values of R_o_.

Finally, simulation results (not included) for changes in the time lag regarding the primary *Exo-erythrocytic Schizogony *(k_in_) show high correlation coefficients and low mean square errors for values of k_in _in the range 8–10 days. For values above or below this threshold, correlation coefficients and mean square errors do not change significantly.

### Sensitivity to initial conditions

Under the assumed first scenario and for a daily survival probability (p) of 0.899, simulation results show that the mathematical model is able, although not incredibly so (R = 0.396 and MSE = 0.0049), to reproduce increasing and decreasing trends observed in malaria incidences. Nevertheless, the model considerably overestimates recorded disease outbreaks. If p is set to 0.890, when temperatures are in the interval (27–30°C] (as shown in Figure 9), R increases to 0.566 and MSE drops to 0.0009. In this case, the model adequately represents the observed malaria incidence for the period from January 1994 to January 2002 and overestimates malaria outbreaks during February-November 2002. For daily survival probabilities below 0.880, when temperatures are above 30°C, the model shows that the malaria disease dies out. For values of p ranging from 0.895 to 0.901, when temperatures are in the favourable range 29–24°C for vector survivorship, R ranges from 0.448 to 0.374, and MSE ranges from 0.0027 to 0.0062.

Under the assumed second scenario and for a feeding interval (FI) of 2.0 days, simulation results show that the mathematical model is not able (R = 0.196 and MSE = 0.0011) to represent seasonality, prevalence and time of occurrence of recorded outbreaks. The model briefly simulates the observed values for the period from January 1994 to June 1999 but significantly overestimates malaria incidence from July 1999 onward. If FI is set to 2.1 days, when temperatures are in the favourable range for vector survivorship (Figure 9), the model adequately represents the observed incidence (R increases to 0.472 and MSE decreases to 0.0001). For values of FI below 2.0 days, i.e. 1.7 days, R dramatically drops to 0.008 and MSE increases to 0.0115. As expected, malaria incidence and outbreaks are significantly overestimated.

Finally, simulation results for the assumed third scenario show that the mathematical model is not able (R = 0.151 and MSE = 0.0042) to represent seasonality, prevalence and time of occurrence of recorded outbreaks. For a constant sporogonic cycle (n) of 8.0 days, the model significantly overestimates malaria incidence throughout the entire simulation period. If n is set to 10 days, when temperatures are in the favourable range for vector survivorship (Figure 9), the model adequately represents the observed disease incidence (R increases to 0.576 and MSE decreases to 0.0001). Finally, for values of n below 8 days, i.e. n = 7 days, R decreases to 0.113 and MSE increases to 0.0082. As expected, malaria incidence and outbreaks are significantly overestimated.

### Final simulation scenarios

**Figure 12:**
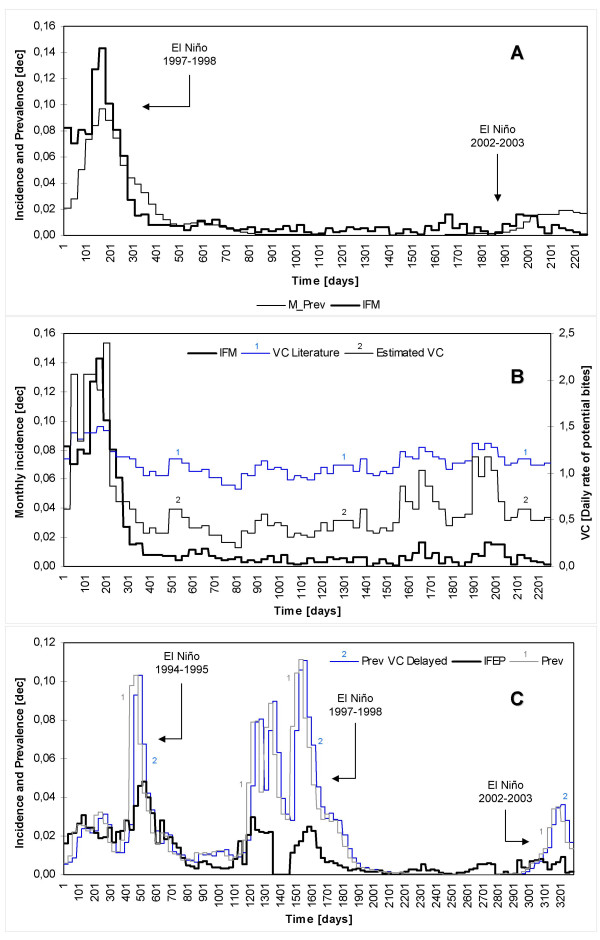
**Time series of model results for final simulation scenario**. (A) IFM denotes the monthly *P. falciparum *malaria incidence (black solid line) observed in Nuqui during the period November 1^st^, 1997-December 31^st^, 2003 (2,252-day simulation period). Variable M_Prev denotes the simulated *monthly *malaria prevalence (gray solid line). (B) Values of the Vectorial Capacity for entomological variables proposed in the literature (see blue solid line 1) and using field-based approximations and/or estimated values under controlled laboratory conditions (black solid line 2). (C) IFEP denotes the *P. falciparum *malaria incidence (black solid line) observed per EP in El Bagre during the period January 3^rd^, 1994 – December 28^th^, 2002 (3,282-day simulation period). Variable Prev denotes the malaria prevalence rate (gray solid line 1) obtained for each EP during the corresponding simulation period. Time series Prev VC Delayed (blue solid line 2) denotes the simulation results for a delayed Vectorial Capacity.

Figure 12 depicts the time series of model results for the Nuqui region when a final simulation scenario is considered. In this case, the entomological variables involved in the estimation of the Vectorial Capacity of *An. albimanus *were replaced by field-based approximations and/or values observed under controlled laboratory conditions (see Figure 12B). Under this final scenario, the mathematical model adequately reproduced (R = 0.897 and MSE = 0.0002) both seasonality and prevalence of *P. falciparum *malaria recorded in this high-endemic area during the period from November 1^st^, 1997 to December 31^st^, 2003.

Figure 12 also depicts the time series of model results for a first final simulation scenario considered for El Bagre region (see solid line 1 in Figure 12C), in which (a) a crude seasonality of mosquito density (values between 9–11 mosquitoes per human host) is assumed, according to the availability of adequate breeding sites; (b) the HBI exogenous variable is set to a constant value of 0.41; (c) the DD exogenous variable is set to 114°C-days; (d) the ν exogenous variable is set to 0.95 years; (e) the k_in _endogenous variable is set to 8 days; (f) the daily survival probability of the mosquito host is set to a constant value of 0.890; and (g) the endogenous variables FI and n are assumed to be function of mean daily temperatures.

Under this final scenario, the proposed mathematical model adequately reproduced (R = 0.628 and MSE = 0.0005) both seasonality and prevalence of *P. falciparum *malaria recorded in the area during the period from January 3^rd^, 1994 (first EP 1994) to December 28^th^, 2002 (13th EP 2002). Noteworthy, is that the system of differential equations was able to replicate malaria outbreaks that were recorded in this endemic prone-region as a result of climatic and environmental anomalies associated with the occurrence of the El Niño in the Tropical Pacific (Figure 12C). The period from January 1994 to December 1996, which comprised the event of the El Niño 1994–1995, was better simulated. Nevertheless, modelling results still exhibit peaks in transmission two epidemiological periods before recorded outbreaks (see solid line 1 in Figure 12C).

Finally, Figure 12C depicts the time series of model results for a second final simulation scenario considered for the El Bagre region, in which the Vectorial Capacity was delayed one epidemiological period (see solid line 2 in Figure 12C). In this case, the correlation coefficient between observed and simulated malaria incidence rates increased to 0.668, whereas the mean square error remained constant.

### Climate scenarios

**Figure 13:**
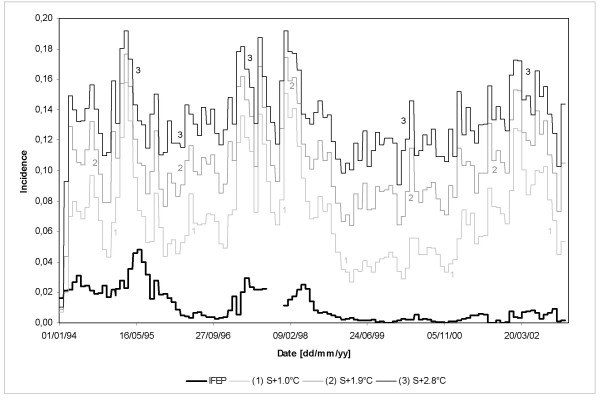
**Time series of model results for climate scenarios**. IFEP denotesthe *P. falciparum *malaria incidence (black solid line) observed per EP in the El Bagre region during the period January 3^rd^, 1994 – December 28^th^, 2002 (3,282-day simulation period). Time series (S_+1.0_), (S_+1.9_) and (S_+2.8_) represent model results for increases of 1.0°C (solid line 1), 1.9°C (solid line 2) and 2.8°C (solid line 3), respectively, in mean daily ambient temperatures.

Figure 13 presents the results for the simulation period from January 3^rd^, 1994 (first EP 1994) to December 28^th^, 2002 (13th EP 2002) under the scenarios (S_+1.0_), (S_+1.6_, not shown), (S_+1.9_) and (S_+2.8_). For a changing climate scenario (S_+1.0_, see solid line 1 in Figure 13), the model indicates that for an increase of +1.0°C in mean daily temperatures and a constant vector density D_0_, malaria reaches an average prevalence of about 7% of the total population at risk. Moreover, peaks in *P. falciparum *malaria prevalence during the El Niño warm events could reach 12.6, 15.1 and even 15.7% of the total number of people living in rural areas (Figure 13). For a S_+1.9 _scenario (an increase of +1.9°C in mean daily outdoor temperatures and a constant vector density D_0_; solid line 2 in Figure 13), model results suggest that malaria prevalence might reach an average value of about 11% of the total population at risk. In this case, peaks in transmission could increase to almost 17.7%. Finally, under the S_+2.8 _scenario, modelling results show an average prevalence of 14% and peaks in transmission during the periods of expected outbreaks reaching 19.2% of the total population (see solid line 3 in Figure 13).

## Discussion

Application of the model during the validation periods from November 1^st^, 1997 to December 31^st^, 2003 and from January 3^rd^, 1994 to December 28^th^, 2002 for the Nuqui and El Bagre regions, produced adequate results, with correlation coefficients and mean square errors between observed and modelled malaria incidences reaching R = 0.897–0.668 (P > 0.95) and MSE = 0.0002–0.0005, respectively. Under the described epidemiological scenarios, modelling results suggest that malaria can develop in the populations at risk at low endemic levels and minor efforts can lead to the eradication of the disease.

For the El Bagre region it was found that a constant density of about 9–10 mosquitoes per human host are required for the development of malaria in the community. The results of the analysis of instability cases seem to suggest that, in order to counteract malaria in the selected region, it is not necessary to eliminate *Anopheles *mosquitoes from there entirely: field campaigns aimed at the control and eradication of the disease must reduce the number of vectors to values below 9 mosquitoes per human host. The model also suggests that if environmental conditions favour the development of the mosquito population (letting vector densities reach values above 11 mosquitoes per human host), malaria disease will propagate in the community and reach endemic levels of 9% of the total population at risk. In this case, malaria outbreaks could affect almost 15% of the total number of people living in the area.

The results of the analysis of changes in Human Blood Index seem to suggest that *An. darlingi*, the common primary vector found in El Bagre, is basically zoophilic in its feeding behaviour. It is of interest to note that, if this characteristic of the vector population drastically increases to 60% of total bites on humans, malaria disease could propagate in the community at high endemic levels and reach 20% of the total population at risk.

Based on modelling results it can therefore be argued that if an infection of *P. falciparum *lasts for a period of time greater than 1.1–1.2 years in humans (when left untreated), malaria could propagate rapidly in the population at risk and reach high-endemic levels. If the average infected period is shortened to periods below 0.9 years, the model suggests that the disease could be controlled through the ecosystems themselves and minor efforts can lead to its eradication. The Human Average Infected Period might be considered one of the critical variables for understanding the malaria cycle in the vertebrate host. Under our conditions in the field (research is still ongoing), most patients are treated immediately or within one month after the detection of malaria parasites in their blood streams.

Simulation results also suggest, as expected, that changes in the time lag representing the Primary *Exo-erythrocytic Schizogony *do not appreciably affect malaria transmission. In fact, the duration of the parasite cycle in human hosts is more dependent on host characteristics than on external ecological factors.

So far, the mathematical model suggests those entomological variables more strongly affected by climatic conditions and that in turn finally impact transmission potential of mosquito population. These parameters include the sporogonic cycle of the malaria parasite, the daily rate of vector biting, the daily rate of vector natality and the daily survival probability of the mosquito host. Field research must be undertaken in order to estimate how changes in microclimatic and environmental conditions of breeding and resting sites affect these relevant entomological variables. Other variables such as hatching, larval developing, and adult emerging time delays, which seem also to be affected by ambient temperatures, must be included as well.

However, simulation results show that the degree days required for digestion of a portion of ingested blood and for parasite development, which in turn influence the feeding interval and the sporogonic cycle inside vectors, respectively, might be the key variables that cause sensitivity to initial conditions and affect or control malaria transmission. According to these results, vector survivorship is NOT the most important element in the Basic Reproduction Rate of malaria. This conclusion is contrary to Macdonald's statement: '*the formula for the Basic Reproduction Rate holds that the influence of vector survivorship is greater than the influence of the average number of men bitten by one mosquito in one day or the sporogonic cycle, which are in turn greater than the influence of the proportion of anophelines with sporozoites in their salivary glands which are actually infective, or the proportion of affected people, who have received one infective inoculum only, who revert to the unaffected state in one day*' [19]. Nevertheless, it has to be said that research is still ongoing.

According to the model and based on the simulated scenarios, malaria outbreaks in the selected regions are possible during the favourable periods following the onset of the El Niño warm event. This epidemiological scenario is primarily controlled by the sporogonic cycle and the feeding interval, which are strongly affected by ambient temperatures. Hence, under the conditions in the field, it appears that temperature becomes the most relevant parameter driving the final malaria incidence and must be considered an essential variable for an adequate representation of malaria transmission in the selected endemic areas. It has to be argued that precipitation, which controls the availability of adequate breeding sites, is responsible for representing observed fluctuations in vector density. Even though mosquito's density is not the key variable for understanding the patterns of malaria outbreaks in the selected region, the model results show that a crude seasonality of vector density is also needed for understanding disease transmission. The model suggests that between periods of high temperatures, malaria disease is maintained at low endemic levels due to the role of vector density. Field research is currently under way in order to consider possible synergistic effects of temperature and precipitation on malaria transmission.

Simulation results suggest that assessing the impact of climate on malaria transmission not only requires consideration of the changes in annual mean temperatures, but also, and more importantly, the extent of inter-annual variability in both temperature and rainfall.

Most of the mathematical models used to derive the standard formulae in malaria epidemiology assume a homogeneous mosquito population, random and uniform vector bites on humans, and a constant adult mosquito population size. Although the first two useful approximations were followed, simple differential equations and basic entomological exogenous variables were included for representing seasonal fluctuations of mosquito density. Thus, the intrinsic differences in mortality rates, the mosquito biting preferences, and the proximity to larval habitats were deliberately ignored. But the dynamics of vectors during pre-imago stages, the predator-prey-food interactions during larval stage, and the relationships between environmental factors and survivorship-behaviour of vectors were purposely included. In fact, the analysis of human biting rates available from field campaigns conducted up to this point shows that, as expected, population densities might be significantly associated with observed rainfall patterns. These vector dynamics, following the preliminary analyses, are being clearly captured by the mathematical model.

New analysis will focus on more detailed examination of key variables that control the dynamic response of malaria transmission in the study sites. Particular interests include testing the model for parsimony and simplicity. A thorough dissection of all the components is currently being conducted in order to capture the most basic interactions between climatic and entomological variables. The mathematical tool will be improved by the inclusion of new subroutines for predator-prey interactions, as well as new field and laboratory data for the entomological parameters.

New analysis will focus also on additional stage variables (following Yang's equations [20,21]) that will be used to describe different levels of protection or anti-malarial immunity, which are commonly observed among people exposed to continuous and intense disease transmission. Migratory patterns and exogenous introduction of infected individuals at certain times are to be included in the model.

## Conclusion

The observed monthly values of malaria incidence are characterized by seasonal oscillations alternating irregularly with high incidence periods. Modelling results must show at least such fluctuations of disease transmission. Simulations using only mean annual temperatures do not represent this characteristic. In contrast, simulations using mean monthly/daily temperatures adequately represent the seasonality of malaria prevalence. Apparently, results seem to suggest that the intra-annual cycle of mean temperatures is good enough to represent the seasonal oscillations of monthly malaria incidence rates in the selected study sites. However, simulation results show that seasonality of vector density also becomes an important factor towards understanding disease transmission, thus suggesting that seasonal and inter-annual variability of both temperature and rainfall are required to assess the impact of climate on malaria dynamics.

In the applied model described here, attempts were made to include the seasonality of vector density. The module representing the availability of breeding sites was intentionally used to estimate the seasonal fluctuations of such an important entomological variable. Modelling the pre-imago cohorts for different larval habitats resulted in an estimation of the availability of reservoirs. The BPAM model simulations suggested that (a) oviposition and the development of mosquitoes during pre-imago stages are significantly slower during dry periods, and (b) egg-laying by the mosquito population is appreciably enhanced during periods of notable availability of adequate larval habitats. The system of differential equations also included the simulation of vector dynamics in the field on a daily time scale. In addition to climate data, biological information was required to incorporate species-specific values for each of the parameters of the mathematical model. As a result, seasonal fluctuations in vector density were predicted, with values in the range of the estimated critical densities necessary to maintain parasite transmission.

Malaria is a complex disease that cannot be studied in a uni-disciplinary and exclusively qualitative-descriptive manner. The research project has used an interdisciplinary approach for understanding malaria epidemiology by studying, concurrently, the mosquito vector ecology, the malaria parasite, the human population dynamics, and the characteristics of our heterogeneous environment. Thus, this effort summarizes the joint work of diverse disciplines including Mathematics, Hydrology, Climatology, Entomology, Epidemiology, Field Study and Public Health Medicine.

The mathematical model and the proposed simulation process constitute promising tools to deepen the understanding of the entomological, epidemiological, and climatic interactions related to malaria transmission conducive to disease outbreaks. Mathematical models could help in comprehending how climatic (and non-climatic) factors affect the dynamics of malaria transmission in order to evaluate its spatial and temporal risks in each endemic area. A profound understanding of all these linkages is required for basic research, and for important practical mitigation and human health control interventions. In this context, biological models are significantly necessary to have a quantitative understanding of malaria transmission. As this model is still in its early stages of development, so far it has not been used for operational purposes. However, in the foreseeable future, biological/eco-epidemiological models could be implemented as powerful tools to diagnose possible dynamic patterns of malaria incidence under several entomological, demographic and climatic scenarios, as well as decision-making tools, for the early detection and control of disease outbreaks.

Transmission models could permit (a) to estimate the time of occurrence of unexpected malaria outbreaks; (b) to evaluate the possible magnitude of the concomitant sharp rises in the incidence of the disease; and (c) to pose and answer "what if" questions for remote areas potentially vulnerable to changing scenarios, for which data is commonly sparse. Mathematical models could also identify those elements or appropriate components of the complex eco-epidemiological systems for which there exist proven and effective interventions, particularly in the areas of mosquito control, education in environmental health, and treatment. That is, comprehensive models could provide quantitative goals for effective interventions adapted to the specific ecological circumstances of each endemic area. Then, mathematical models will be able to determine the most appropriate preventative actions that have to be taken in order to reduce the vulnerability of populations to climate-induced epidemics and to prevent outbreaks before they begin. Finally, they are also able to identify 'when' these interventions need to be implemented in each area.

Although malaria is a highly complex multi-factorial disease related to diverse socio-economic and demographic factors, it has been demonstrated here that environmental factors and climate variability go a considerable way in explaining fluctuations of disease incidence in these particular endemic areas. Forecasts of relevant climatic variables (future ENSO events) can be merged with selected mathematical tools (statistical and biological/eco-epidemiological models), creating a Malaria Early Warning System (MEWS) to facilitate early, coupled and environmentally sound public health interventions [8].

A complete MEWS framework will permit a continuous evaluation of the local risk of malaria transmission in the face of multiple changing scenarios. The MEWS could then be combined with several public health strategies in order to develop Integrated Malaria Surveillance and Control Systems. Other components should be included, such as: (a) epidemiological surveillance and control activities, (b) early diagnosis and treatment of primary cases, and (c) entomological surveillance and control activities.

## Authors' contributions

DR developed and implemented the mathematical model, analysed malaria prevalence in the El Bagre region, processed the epidemiological and hydrological time series, carried out the homogeneity analysis, performed the correlation analysis, carried out the experimentation-validation analysis, and drafted the manuscript. GP conceived the first modelling study and participated in its design and coordination. GP, IDV, MLQ, and GLR participated in the validation-analysis process and helped to draft the document. IDV, MLQ, GLR, LEV, and JSZ monitored malaria incidence in the Nuqui region and carried out the entomological analysis. All the authors read and approved the final manuscript.
